# Geranylgeraniol Prevents Statin-Dependent Myotoxicity in C2C12 Muscle Cells through RAP1 GTPase Prenylation and Cytoprotective Autophagy

**DOI:** 10.1155/2018/6463807

**Published:** 2018-05-21

**Authors:** Anna Jaśkiewicz, Beata Pająk, Anna Litwiniuk, Kaja Urbańska, Arkadiusz Orzechowski

**Affiliations:** ^1^Department of Physiological Sciences, Warsaw University of Life Sciences (SGGW), Nowoursynowska 159, 02-776 Warsaw, Poland; ^2^Independent Laboratory of Genetics and Molecular Biology, Kaczkowski Military Institute of Hygiene and Epidemiology, Kozielska 4, 01-163 Warsaw, Poland; ^3^Department of Neuroendocrinology, Centre of Postgraduate Medical Education, Marymoncka 99/103, 01-813 Warsaw, Poland; ^4^Department of Morphological Sciences, Warsaw University of Life Sciences (SGGW), Nowoursynowska 159, 02-776 Warsaw, Poland

## Abstract

The present study investigated the cytotoxic effects of statins (atorvastatin (ATR) and simvastatin (SIM), resp.) and methyl-beta-cyclodextrin (M*β*CD), at their respective IC_50_ concentrations, on muscle regeneration in the in vitro model of murine C2C12 myoblasts. Cotreatment with mevalonate (MEV), farnesol (FOH), geranylgeraniol (GGOH), or water-soluble cholesterol (Chol-PEG) was employed to determine whether the statin-dependent myotoxicity resulted from the lower cholesterol levels or the attenuated synthesis of intermediates of mevalonate pathway. Our findings demonstrated that while GGOH fully reverted the statin-mediated cell viability in proliferating myoblasts, Chol-PEG exclusively rescued M*β*CD-induced toxicity in myocytes. Statins caused loss of prenylated RAP1, whereas the GGOH-dependent positive effect was accompanied by loss of nonprenylated RAP1. Geranylgeranyltransferases are essential for muscle cell survival as inhibition with GGTI-286 could not be reversed by GGOH cotreatment. The increase in cell viability correlated with elevated AKT 1(S463) and GSK-3*β*(S9) phosphorylations. Slight increase in the levels of autophagy markers (Beclin 1, MAP LC-3IIb) was found in response to GGOH cotreatment. Autophagy rose time-dependently during myogenesis and was inhibited by statins and M*β*CD. Statins and M*β*CD also suppressed myogenesis and neither nonsterol isoprenoids nor Chol-PEG could reverse this effect. These results point to GGOH as the principal target of statin-dependent myotoxicity, whereas plasma membrane cholesterol deposit is ultimately essential to restore viability of M*β*CD-treated myocytes. Overall, this study unveils for the first time a link found between the GGOH- and Chol-PEG-dependent reversal of statin- or M*β*CD-mediated myotoxicity and cytoprotective autophagy, respectively.

## 1. Introduction

Atherosclerosis and cardiovascular diseases are main causes of mortality in humans [1]. Statins, which act as 3-hydroxy-3-methylglutaryl coenzyme A reductase (HMG-CoAR; EC 1.1.1.88) inhibitors, are efficient antihyperlipidemic medications and, through their capability of decreasing the intracellular synthesis of cholesterol, act as highly successful cholesterol-lowering drugs [2, 3]. Despite exhibiting various pleiotropic beneficial effects including anti-inflammatory, antithrombogenic, antisclerotic, antiosteoporotic, and anticancer properties [4], some varieties of statins induce skeletal muscle injury including myalgia, myositis, and life-threatening rhabdomyolysis [5]. The mechanism(s) leading to statin myotoxicity is unknown although existing data indicate the association of the drugs with vacuolation of skeletal muscle fibers, blebbing of sarcolemma and cell necrosis [6]. It is therefore imperative to ascertain the etiology of statin-dependent myopathy to preserve normal skeletal muscle function during treatment or at least to be able to improve the regeneration/healing process of injured muscle. Geranylgeraniol (GGOH) seems a good candidate as few reports showed the beneficial role of the compound in rescuing statin-induced myopathy [6, 7] although work by other authors has questioned the causal relationship between muscle safety and protein isoprenylation [8]. In spite of the beneficial effects of GGOH, the molecular mechanisms behind its cytoprotective action remain to be elucidated.

The RAS subfamily of small molecular weight GTPases—active when bound to GTP and inactive in the GDP-bound state [9, 10]—is known to regulate many physiological responses including cell adhesion and growth, apoptosis, cytoskeleton remodeling, motility, and intracellular vesicular transport [11, 12]. Cellular regulatory proteins include both positive (guanine nucleotide exchange factors (GEFs)) and negative regulators (GTPase-accelerating proteins (GAPs)). Hamartin (TSC1) and tuberin (TSC2) proteins act together (TSC1/TSC2) to inhibit muscle growth [13]. Furthermore, native tuberin contains an activity that specifically stimulates the intrinsic GTPase activity (inactivation) of RAP1A but not RAP2, H-RAS, RAC, or RHO [14]. Thus, if RAP1A stimulates muscle growth through mTORC1 (mammalian target of rapamycin complex 1), its activity is repressed by tuberin acting as GAP, which in turn is inhibited by PI3-K/AKT signaling pathway [15]. It should be stressed that GSK-3*β*, downstream substrate inactivated by AKT1, activates TSC1/TSC2. Altogether, activation of PI3-K/AKT signaling should indirectly (by inhibiting TSC1/TSC2 and GSK-3*β*) stimulate RAP1A activity.

There is evidence that statin-induced muscle toxicity is associated with inhibition of protein geranylgeranylation [16]. Prenylation with nonsterol isoprenoids is an essential step in activating certain small GTPases including RAP. This reaction is solely dependent on geranylgeranylation of RAP1A catalysed in skeletal muscle by prenyltransferases including protein geranylgeranyltransferase type I, EC 2.5.1.59, and protein geranylgeranyltransferase type II, EC 2.5.1.60 [17].

Skeletal muscle growth and regeneration (myogenesis) take place in two subsequent phases. After an initial stage of cell proliferation, myoblasts withdraw from the cell cycle and differentiate and fuse to form multinucleated myotubes and muscle fibers [18, 19]. The exact role of RAP1A in each one of these steps is not known, although some studies conducted with RAP1A overexpressing muscle cells indicate that while GTP-bound RAP1A inhibits myogenic differentiation, the GDP-bound protein favors the formation of myotubes [20]. Previous studies from the same group also indicate that RAP1A may regulate the structural organization of late endosomes and lysosomes and therefore influence intracellular degradative pathways [21]. In this sense, GDP-bound RAP1A clustered with acidic structures in the perinuclear region of the myoblasts where the lysosomal proteolytic enzymes likely play an important role during myogenic differentiation [22]. Current research also points out to autophagy as an important tool supporting the intracellular reorganization processes needed for conversion of myoblasts to myotubes [23].

Taken together, the aim of our study was to bridge the gap of understanding molecular mechanism of statin-dependent myopathy.

## 2. Materials and Methods

### 2.1. Reagents and Antibodies

Atorvastatin, a cell-permeable, highly potent, and competitive inhibitor of 3-hydroxy-3-methylglutaryl coenzyme A (HMG-CoA) reductase, was purchased from Calbiochem, Merck-Millipore (Darmstadt, Germany). Simvastatin, (R)-mevalonic acid lithium salt, all-*trans*-geranylgeraniol (GGOH), farnesol (FOH), ubiquinol (UBOH), methyl-beta-cyclodextrin (M*β*CD), poly(ethylene-glycol 600)-cholesterol conjugate (Chol-PEG), poly(ethylene-glycol 600) (PEG), 3-(4,5-dimethylthiazol-2-yl)-2-5-diphenyltetrazolium bromide (MTT), bovine serum albumin (BSA), and acridine orange were purchased from Sigma Aldrich, Saint Louis, MO, USA. C-100-Dolichol (Dolichol 20, DOH) was obtained from Larodan, Karolinska Institutet Science Park, Solna, Sweden, and bisbenzimide fluorochrome from Molecular Probes, Eugene, OR, USA.

### 2.2. Cell Cultures

The murine skeletal muscle cell line C2C12 [24] was obtained from the European Collection of Animal Cell Cultures (ECAAC, Salisbury, UK). The cells were initially cultured in growth media (GM), constituted by Dulbecco's Modified Eagle's Medium (DMEM, Biowest, Nuaillé, France), supplemented with 10% (*v*/*v*) heat-inactivated fetal bovine serum (FBS, Gibco, Carlsbad, CA, USA), penicillin/streptomycin (Life Technologies/Thermo Fisher Scientific, Waltham, MA, USA; 50 IU/mL/50 *μ*g/mL), gentamicin sulfate (Sigma Aldrich, Saint Louis, MO, USA; 20 *μ*g/mL), and Fungizone/amphotericin B (Thermo Fisher Scientific, Waltham, MA, USA; 1 *μ*g/mL), and grown until 70–80% confluence. Spontaneous differentiation by growth factor withdrawal was induced replacing growth medium with differentiation medium (DM) consisting of DMEM supplemented with 2% (*v*/*v*) heat-inactivated horse serum (HS, Gibco, Carlsbad, USA) and the same antibiotic/antimycotic cocktail as the GM and further incubation for up to 5 days. Muscle cells and syncytia were harvested on days 1, 3, and 5 of myogenic differentiation time points at which they displayed different phenotypes. At day 1, cells exhibited proliferating myoblasts, at day 3, differentiating myotubes, and at day 5, differentiated myotubes.

Treatment of the cultures with the different experimental factors tested in the current work was performed 24, 72, or 120 hours prior to cell harvesting based on the respective half-maximal inhibitory concentrations (IC_50_, Supplementary data 1). Statins (ATR or SIM) were administered at various concentrations during differentiation: ATR: day 1—100 *μ*M, day 3—46 *μ*M, and day 5—36 *μ*M; SIM: day 1—125 *μ*M, day 3—10 *μ*M, and day 5—7.5 *μ*M. M*β*CD was added at 2.7 mM, 1.9 mM, and 1.1 mM final concentrations at day 1, day 3, and day 5, respectively.

Statin concentrations seem to be irrelevant regarding the plasma concentrations observed in mice and in humans (nanomolar). The discrepancy is explained by the time of experiment which lasted merely 5 days (in vivo myogenesis lasts considerably longer—at least few weeks). As reported by us, the IC_50_ concentration of statins is inversely proportional to the time of treatment.

### 2.3. Assessment of Cell Viability

Cell viability was assessed evaluating the ability of cells to convert soluble MTT (3-(4,5-dimethylthiazol-2-yl)-2-5-diphenyltetrazolium bromide) into an insoluble purple formazan as described [25]. Briefly, cells grown and differentiated as above were incubated for 4 h at 37°C with MTT (0.5 mg/mL in DMEM without phenol red). Water-insoluble formazan was immediately dissolved in DMSO (100 *μ*L per well) and color evaluated at 570 nm with TECAN 200 multiplate reader (TECAN, Austria).

### 2.4. Vital Staining with Acridine Orange

C2C12 muscle cells, seeded in 24-well culture plates (Corning-Costar, Sigma Aldrich, Saint Louis, MO, USA) and treated with the experimental factors for 1–5 days as above, were subjected to vital staining with acridine orange (AO) and evaluated for morphological changes. Briefly, cell cultures were incubated for 10 min at 37°C (humidified 5% CO_2_/95% air incubator) with AO (1 *μ*g/mL) and HO33342 (10 *μ*g/mL), both dissolved in DMEM without phenol red, and analyzed in an Olympus IX-71 inverted fluorescence microscope. Abundance of AVO was calculated as red to green fluorescence intensity ratio (R/G-FIR) in each microscopic field, as previously described [26]. At least 10 replicates for each treatment as well as nontreated control cells were quantitated using the Cell^F software platform (Olympus Camera, Tokyo, Japan) and Fiji (ImageJ) open-source image processing package [27].

### 2.5. Immunocytofluorescence and Evaluation of Myotube Index

Myotube index and the distribution and localization of total sarcomeric myosin heavy chain (MyHC) were evaluated by immunocytofluorescence (ICF). C2C12 myoblasts were seeded onto 8-chamber slides (0.8 cm^2^/well; Lab-Tek, Thermo Fischer Scientific, Rochester, NY, USA) and cultured in the presence or absence of the different metabolic inhibitors, as above. At selected time points (1, 3, and 5 days), the cells were fixed (1% formaldehyde, 15 min, RT) and permeabilized by subsequent incubation with 0.5% Triton X-100 (10 min, RT) and 70% ethanol (10 min, 4°C). After blocking with PBS containing 1% BSA and 5% normal donkey serum (NDS; 30 min, RT), the cells were subsequently incubated with mouse monoclonal anti-MyHC antibody, clone MF-20 (Developmental Studies Hybridoma Bank, University of Iowa, Iowa City, IA, USA; 1 : 30 in 1% BSA-PBS, for 30 min, RT), and Alexa Fluor 488-conjugated donkey antimouse IgG (AffiniPure F(ab')_2_ fragment Jackson ImmunoResearch Laboratories Inc., West Grove, PA, USA; 1 : 50 in 1% BSA-PBS). The nuclei were visualized by staining with bisbenzimide (Hoechst 33342, Molecular Probes Inc., Eugene, OR, USA; 10 *μ*g/mL, 30 min, 4°C). As negative control, primary antibody was replaced by 1% BSA/PBS. In all cases, slides were mounted with mounting medium (Dako/Agilent Technologies, Santa Clara, CA, USA or Mowiol, Calbiochem-Novabiochem Co. La Jolla, CA, USA) and fluorescence was evaluated in an Olympus BX-60 microscope.

Myotube index was calculated based on cell morphology assessed by contrast phase and the ICF evaluation of Hoechst-stained cells in 10 randomly selected fields. The index was determined as the ratio of the number of myotubes—defined as cells containing at least three nuclei—to the total number of nuclei multiplied by 100%.

### 2.6. Apoptotic Index

The presence of apoptotic nuclei, assessed by nuclear shrinkage and chromatin condensation, was evaluated using Cell^F software platform (Olympus Camera, Tokyo, Japan) and Fiji (ImageJ) open-source image processing package [27]. Ten microscopic fields representing at least 100 cells for each treatment were evaluated, and apoptotic index (AI) was calculated as the percentage of apoptotic nuclei/total number of nuclei in at least 10 replicates for each treatment and nontreated controls.

### 2.7. Western Blot Analysis

The cells, after the different treatments described above, were lysed in RIPA buffer (10 mM Na_4_P_2_O_7,_ 50 mM HEPES, 150 mM NaCl, 1% triton-X, 0.1% SDS, 10 mM EDTA, and 100 mM NaF, pH 7.4) containing protease inhibitor cocktail (Roche, Mannheim, Germany), insoluble material removed by centrifugation (10,000*g*, 5 min), and protein levels quantitated with Bradford reagent (Bio-Rad, Hercules, CA, USA). Each protein extract was adjusted to a 2 *μ*g/*μ*L concentration, added with Laemmli sample buffer (4x concentrate, Bio-Rad, Hercules, CA, USA), heated for 5 min at 95°C, and separated on precast polyacrylamide gradient gels (7.5–12%). After transfer to 0.2 *μ*M polyvinylidene difluoride membranes (PVDF, Bio-Rad), the membranes were blocked in either 5% (*w*/*v*) nonfat dried milk or 1% (*w*/*v*) BSA (Sigma Aldrich), diluted in tris-buffered saline containing 0.1% (*v*/*v*) Tween 20 (TBS-T), and probed overnight at 4°C with the respective primary antibodies, as follows: T-AKT1 (C-20, Santa Cruz Biotechnology, Dallas, CO, USA), P-AKT1 (Ser473) (Santa Cruz Biotechnology, Dallas, CO, USA), T-GSK-3*β* (H-76, Santa Cruz Biotechnology, Dallas, CO, USA), P-GSK-3*β* (Ser9) (Santa Cruz Biotechnology, Dallas, CO, USA), actin (C-11, Santa Cruz Biotechnology, Dallas, CO, USA), Beclin 1 (H-300, Santa Cruz Biotechnology, Dallas, CO, USA), RAP1A (C-17, Santa Cruz Biotechnology, Dallas, CO, USA), RAP1A/1B (26B4, Cell Signaling Technology, Danvers, MA, USA), RAP1B (36E1, Cell Signaling Technology, Danvers, MA, USA), MAP LC3-Ib/MAP LC3-IIb (Cell Signaling Technology, Danvers, MA, USA), T-MyHC (MF-20, DSHB (Developmental Studies Hybridoma Bank), IA, USA), and myogenin (F5D, DSHB (Developmental Studies Hybridoma Bank), IA, USA). The membranes were subsequently incubated with the respective HRP-linked antirabbit and antimouse IgG (Cell Signaling Technology, Danvers, MA, USA) or antigoat (Cell Signaling Technology, Danvers, MA, USA) antibodies and developed by ECL (Pierce™ ECL Western Blotting Substrate, Thermo Fisher Scientific). Densitometric quantification of band intensities was performed using analysis software (Image Studio Lite Version 5.2.5, LI-COR Biotechnology—GmbH, Bad Homburg, Germany) and the open-source image processing package Fiji (ImageJ).

Differences in the phosphorylation state of specific proteins were determined probing the Western blot membranes with primary antibodies to the respective phosphorylated forms AKT1 (P-AKT1 (Ser473)) and GSK-3*β* (P-GSK-3*β* (Ser9)) in comparison to the total protein expression levels of the pertinent proteins (AKT1 (T-AKT1) and GSK-3*β* (T-GSK-3*β*)). Levels of Beclin-1, RAP1A (nonprenylated (NP)), RAP1A, RAP1B, RAP1A/B (prenylated (P) and both prenylated and nonprenylated forms (P + NP)), MAP LC3-Ib/MAP LC3-IIb, myogenin, MyHC, and actin were carried out probing the cell lysates obtained at each selected step of myogenesis with the pertinent antibodies.

## 3. Results

### 3.1. Effect of Statins on Cell Viability and Rescue of Statin-Dependent Cytotoxicity in Proliferating Myoblasts by Geranylgeraniol

**Figure 1:**
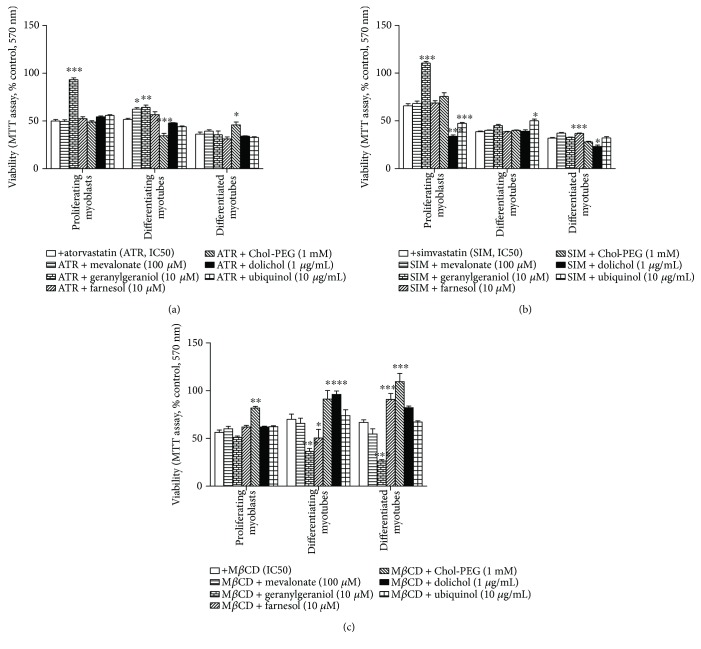
Effect of nonsterol isoprenoids and soluble cholesterol treatments on C2C12 muscle cell viability. Nonsterol isoprenoids and soluble cholesterol differentially rescue C2C12 myoblasts from statin- or M*β*CD-reduced (IC_50_) cell viability. C2C12 myoblasts were exposed for 24, 72, or 120 h to statins or M*β*CD (IC_50_), (day 1—proliferating myoblasts, day 3—differentiating myotubes, and day 5—differentiated myotubes). ATR or SIM was administered in the listed concentration at each day of differentiation: ATR: day 1—76 *μ*M, day 3—46 *μ*M, and day 5—36 *μ*M; SIM: day 1—87 *μ*M, day 3—6 *μ*M, and day 5—3 *μ*M. M*β*CD was added to give the final concentration: day 1—2.7 mM, day 3—1.9 mM, and day 5—1.1 mM or vehicle control (0.1% DMSO or 2% HS DMEM) without or with selected mevalonate pathway intermediate (mevalonate 100 *μ*M, geranylgeraniol 10 *μ*M, farnesol 10 *μ*M, Chol-PEG 1 mM, dolichol 1 *μ*g/mL, and ubiquinol 10 *μ*g/mL). (a) Geranylgeraniol (GGOH, 10 *μ*M) in proliferating myoblasts and differentiating myotubes, farnesol (FOH, 10 *μ*M) in differentiating myotubes, and Chol-PEG in differentiated myotubes inhibited ATR-dependent drop in cell viability. (b) Geranylgeraniol (GGOH, 10 *μ*M) in proliferating myoblasts, ubiquinol in differentiating myotubes, and farnesol in differentiated myotubes inhibited SIM-dependent drop in cell viability. (c) Soluble cholesterol (Chol-PEG, 1 mM) in proliferating myoblasts, differentiating myotubes, and differentiated myotubes; dolichol (DOH, 1 *μ*g/mL) in differentiating myotubes; and mevalonate (MEV, 100 *μ*M) in differentiated myotubes inhibited M*β*CD-dependent drop in cell viability. Two-way ANOVA test [time (proliferating myoblasts, differentiating myotubes, differentiated myotubes)] amounted to *F*_(2,187)_ = 201.73, *p* < 0.0001 for ATR; *F*_(2,227)_ = 665.22, *p* < 0.0001 for SIM; *F*_(2,371)_ = 8.58, *p* < 0.0002 for M*β*CD. Treatment: *F*_(6,187)_ = 38.96, *p* < 0.0001 (ATR, ATR + MEV, ATR + GGOH, ATR + FOH, and ATR + Chol-PEG); *F*_(6,227)_ = 56.33, *p* < 0.0001 (SIM, SIM + MEV, SIM + GGOH, SIM + FOH, and SIM + Chol-PEG); *F*_(6,371)_ = 58.43, *p* < 0.0001 (M*β*CD, M*β*CD + MEV, M*β*CD + GGOH, M*β*CD + FOH, and M*β*CD + Chol-PEG). Interaction: *F*_(12,187)_ = 30.26, *p* < 0.0001 for ATR; *F*_(12,227)_ = 49.40, *p* < 0.0001 for SIM; *F*_(12,227)_ = 11.29, *p* < 0.0001 for M*β*CD. Error bars = SEM and ^∗^*p* < 0.05, ^∗∗^*p* < 0.01, and ^∗∗∗^*p* < 0.001 for comparison with nontreated control cells. Results are means ± SEM of three independent experiments.

Statins are known inhibitors of HMG-CoA reductase (3-hydroxy-3-methylglutaryl-coenzyme A reductase, HMGCR), the rate-controlling enzyme of the mevalonate pathway. Consequently, HMG-CoA reductase inhibitors limit mevalonate availability for several intermediary metabolic pathways including synthesis of nonsterol isoprenoids (geranylgeraniol, farnesol, dolichol, and ubiquinol). The effect of the different experimental factors tested herein (mevalonate (MEV), geranylgeraniol (GGOH), farnesol (FOH), dolichol (DOH), ubiquinol (UBOH), and cholesterol as water-soluble conjugated Chol-PEG) in cell cultures challenged with statins or M*β*CD at their half-maximal cell viability inhibitory concentration (Supplementary data 1) was monitored via the MTT assay at days 1, 3, and 5 of myogenesis.  Figure 1 illustrates the cytoprotective effect of mevalonate pathway intermediates. Full reversal of ATR- and SIM-induced cytotoxicity in proliferating myoblasts was observed after addition of 10 *μ*M GGOH (Figures 1(a) and 1(b), *p* < 0.001). As anticipated, a different pattern of response was observed between differentiating and already differentiated myotubes. While both MEV (100 *μ*M) and GGOH significantly rescue the detrimental effect of ATR on differentiating myotubes (*p* < 0.05), none of them were able to rescue ATR-mediated toxicity in differentiated myotubes. Neither FOH (10 *μ*M) nor UBOH (10 *μ*g/mL) affected ATR-dependent cytotoxicity at any stage of muscle cell differentiation. Chol-PEG (1 mM) exerted a differential effect on ATR-induced toxicity depending on the degree of differentiation. While it potentiates the detrimental effect of ATR in cell viability in differentiated myotubes (*p* < 0.05), the compound rescues the statin effect in differentiated myotubes (*p* < 0.05).

A different pattern was observed in the case of SIM-induced cytotoxicity (Figure 1(b)). GGOH was capable of rescuing toxicity only in proliferating myoblasts and MEV was inefficient independently of the differentiation state. DOH (1 *μ*g/mL) and UBOH (10 *μ*g/mL) exacerbated the SIM-reduced cell viability in proliferating myoblasts (Figure 1(b), *p* < 0.001), while only UBOH improved SIM-reduced cell viability in differentiating myotubes while FOH in differentiated myotubes. FOH was able to rescue SIM-induced toxicity only in differentiated myotubes (*p* < 0.001).

**Figure 2:**
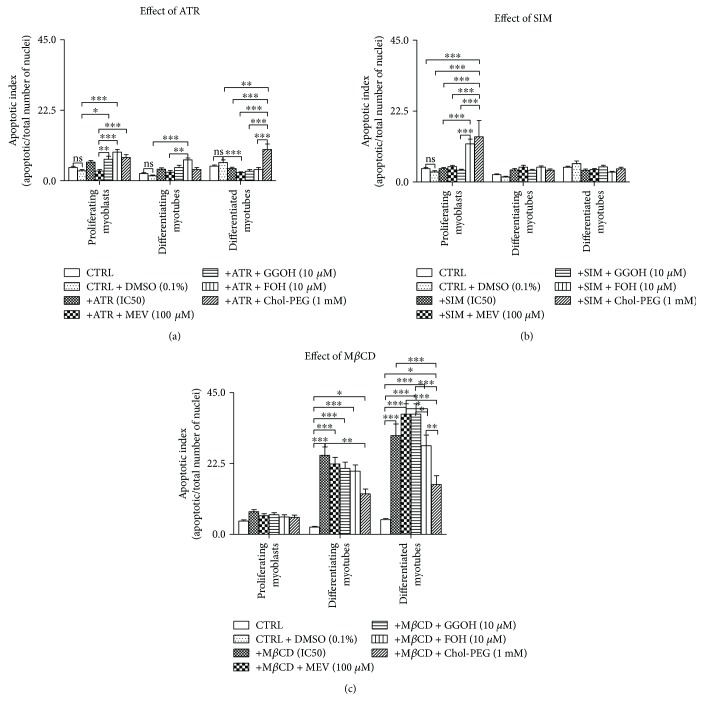
Effect of nonsterol isoprenoids and soluble cholesterol treatments on apoptotic index (AI) in C2C12 myoblasts affected by statins or M*β*CD. C2C12 myoblasts were exposed for 24, 72, or 120 h to statins or M*β*CD (IC_50_), (day 1—proliferating myoblasts, day 3—differentiating myotubes, and day 5—differentiated myotubes). ATR or SIM was administered in the listed concentration at each day of differentiation: ATR: day 1—76 *μ*M, day 3—46 *μ*M, and day 5—36 *μ*M; SIM: day 1—87 *μ*M, day 3—6 *μ*M, and day 5—3 *μ*M. M*β*CD was added to give the final concentration: day 1—2.7 mM, day 3—1.9 mM, and day 5—1.1 mM or vehicle control (0.1% DMSO or 2% HS DMEM) without or with selected mevalonate pathway intermediate (mevalonate 100 *μ*M, geranylgeraniol 10 *μ*M, farnesol 10 *μ*M, and Chol-PEG 1 mM). Next, cells were subjected to vital staining with HO33342 (see Materials and Methods). Apoptotic index (AI) was calculated as percent value of apoptotic nuclei/total number of nuclei in at least 10 replicates for each treatment and nontreated controls. Two-way ANOVA test for AI followed by Bonferroni's multiple comparisons was employed to analyze the data. The results of [time (proliferating myoblasts, differentiating myotubes, differentiated myotubes)] amounted to *F*_(2,189)_ = 17.46, *p* < 0.0001 for ATR; *F*_(2,189)_ = 12.17, *p* < 0.0001 for SIM; *F*_(2,162)_ = 142.3, *p* < 0.0001 for M*β*CD. Treatment: ATR, ATR + MEV, ATR + GGOH, ATR + FOH, and ATR + Chol-PEG (*F*_(6,189)_ = 15.52, *p* < 0.0001), SIM, SIM + MEV, SIM + GGOH, SIM + FOH, and SIM + Chol-PEG (*F*_(6,189)_ = 4.712, *p* = 0.0002), M*β*CD, M*β*CD + MEV, M*β*CD + GGOH, M*β*CD + FOH, and M*β*CD + Chol-PEG (*F*_(5,162)_ = 37.52, *p* < 0.0001). Interaction: *F*_(12,189)_ = 7.629, *p* < 0.0001 for ATR; *F*_(12,189)_ = 5.111, *p* < 0.0001 for SIM; *F*_(10,162)_ = 9.708, *p* < 0.0001 for M*β*CD. Error bars = SEM and ^∗^*p* < 0.05, ^∗∗^*p* < 0.01, ^∗∗∗^*p* < 0.001 for comparison between the means. Results are means of three independent experiments.

To gain insight into the cellular pathways translating into the reduced cell viability depicted in Figures 1(a) and 1(b), the apoptotic index (AI) was calculated based on the analysis of nuclei morphology depicted in the micrographs illustrated in Supplementary data 2. As can be observed from the bar charts, ATR did not modify the value of AI with regard to nontreated control cells (Figure 2(a)). GGOH and FOH at day 1, FOH at day 3, while Chol-PEG at day 5 significantly raised AI versus the nontreated controls (Figure 2(a)). SIM could hardly affect AI, but at day 1, FOH and Chol-PEG significantly elevated a fraction of apoptotic cells (Figure 2(b)).

### 3.2. Effect of M*β*CD on Muscle Cell Viability and Rescue of Cytotoxicity by Chol-PEG, DOH, and MEV

In difference to statins, M*β*CD is a cholesterol chelator affecting membrane nanodomains or lipid rafts, important platforms for cellular signaling downstream to ligand/receptor interactions. In turn, Chol-PEG is a soluble form of cholesterol capable of restoring normal levels of cholesterol in affected membranes. This effect of Chol-PEG was clearly visible in experiments in which M*β*CD was administered to our cell culture paradigm to reduce viability of myoblasts and myotubes. Independent of the time frame at which IC_50_ concentrations of M*β*CD caused cytotoxicity, the addition of Chol-PEG (1 mM) efficiently restored cell viability to levels of control untreated muscle cells (Figure 1(c)). Interestingly, DOH (1 *μ*g/mL) and MEV (100 *μ*M) attenuated M*β*CD effect similarly to Chol-PEG in differentiating and differentiated myotubes (*p* < 0.001). The highest AI values were found after 3- and 5-day treatment with M*β*CD (Figure 2(c), *p* < 0.001). Neither MEV, GGOH, FOH, nor Chol-PEG significantly reduced the percentage of apoptotic cells, albeit Chol-PEG seemed the most efficient.

### 3.3. Statin- and M*β*CD-Dependent Decline in Muscle Cell Viability Are Associated with Altered AKT/GSK-3*β* Signaling Pathway

**Figure 3:**
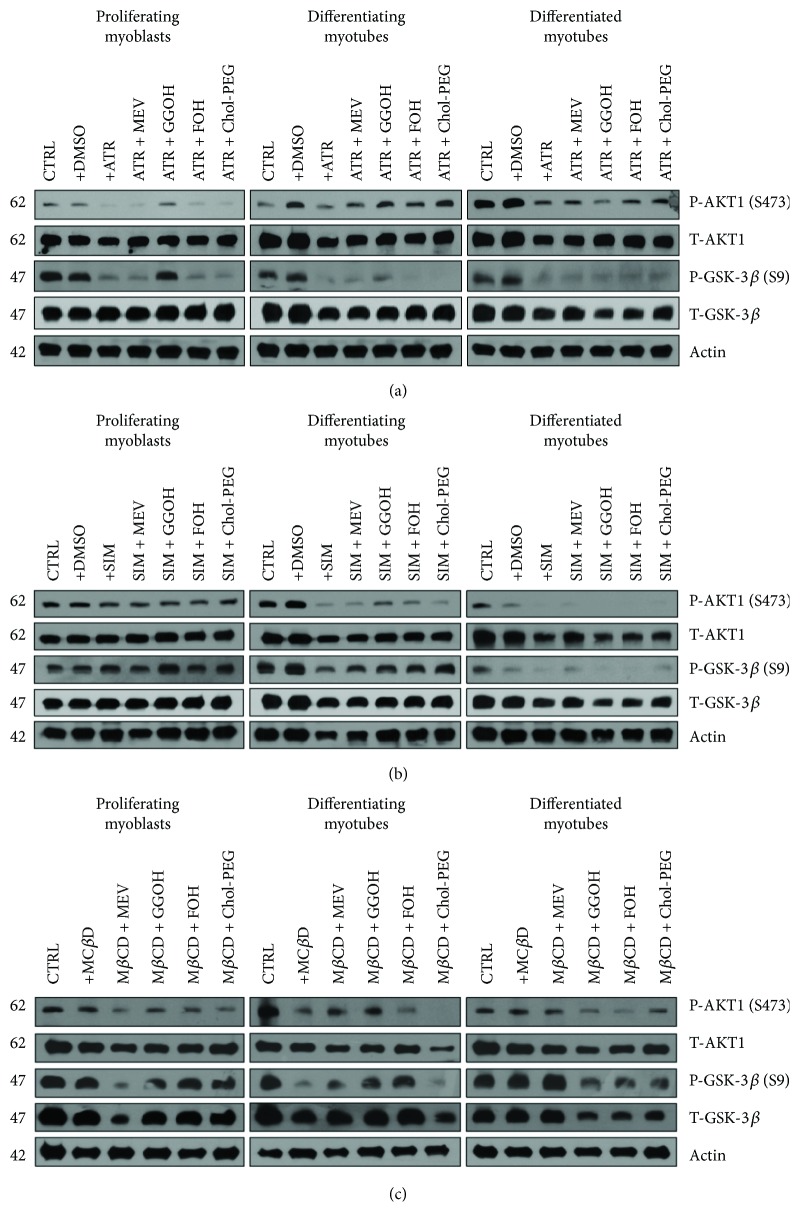
Effect of nonsterol isoprenoids and soluble cholesterol treatments on AKT/GSK-3*β* in C2C12 myoblasts affected by statins or M*β*CD.

IC_50_ concentrations of statins and M*β*CD caused decrease in AKT phosphorylation at serine 473 (P-AKT1-S473), a feature paralleled by diminished GSK-3*β* phosphorylation at serine 9 (P-GSK-3*β*-S9). The AKT/GSK-3*β* cascade plays a fundamental role in muscle cell viability [37] in which P-GSK-3*β*-S9 acts as a substrate for active P-AKT1-S473 (the lower the P-AKT1-S473, the lower the P-GSK-3*β*-S9 expression levels). Noticeably, the changes in cell viability illustrated in Figure 1 correlate with the AKT/GSK-3*β* protein expression levels (Figure 3). Total protein was extracted from differentiating C2C12 myoblasts exposed for 24, 72, or 120 h to statins or M*β*CD (IC_50_) (day 1—proliferating myoblasts, day 3—differentiating myotubes, and day 5—differentiated myotubes). ATR or SIM was administered in the listed concentration at each day of differentiation: ATR: day 1—76 *μ*M, day 3—46 *μ*M, and day 5—36 *μ*M; SIM: day 1—87 *μ*M, day 3—6 *μ*M, and day 5—3 *μ*M. M*β*CD was added to give the final concentration: day 1—2.7 mM, day 3—1.9 mM, and day 5—1.1 mM or vehicle control (0.1% DMSO or 2% HS DMEM) without or with selected mevalonate pathway intermediate (mevalonate 100 *μ*M, geranylgeraniol 10 *μ*M, farnesol 10 *μ*M, and Chol-PEG 1 mM). (a) Atorvastatin (ATR, IC_50_) diminished protein expression levels of P-AKT1-S473 (AKT1 active form) and its substrate GSK-3*β* (P-GSK-3*β*-S9) during in vitro myogenesis. Mevalonate (MEV, 100 *μ*M), geranylgeraniol (GGOH, 10 *μ*M), farnesol (FOH, 10 *μ*M), and Chol-PEG (1 mM) inhibited ATR-dependent drop in P-AKT1-S473/P-GSK-3*β*-S9 protein expression levels in differentiating myotubes. Nonsterol isoprenoids and Chol-PEG could not overturn the ATR effect observed in differentiated myotubes. (b) Simvastatin (SIM, IC_50_) diminished protein expression levels of P-AKT1-S473 (AKT1 active form) and its substrate GSK-3*β* (P-GSK-3*β*-S9) in differentiating and differentiated myotubes during in vitro myogenesis. Geranylgeraniol (GGOH, 10 *μ*M) and to some extent farnesol (FOH, 10 *μ*M) inhibited SIM-dependent drop in P-AKT1-S473/P-GSK-3*β*-S9 protein expression levels in differentiating myotubes. This effect was neither observed in proliferating myoblasts nor in differentiated myotubes. (c) Methyl-beta-cyclodextrin (M*β*CD, IC_50_) diminished protein expression levels of P-AKT1-S473 (AKT1 active form) and its substrate GSK-3*β* (P-GSK-3*β*-S9) in differentiating myotubes during in vitro myogenesis. Mevalonate (MEV, 100 *μ*M), geranylgeraniol (GGOH, 10 *μ*M) and to some extent farnesol (FOH, 10 *μ*M) reversed in part M*β*CD-dependent drop in P-AKT1-S473/P-GSK-3*β*-S9 protein expression levels in differentiating myotubes. Chol-PEG could not overturn the M*β*CD repressive action in differentiating myotubes. A representative blots are shown in (a), (b), and (c) from three independent repeats. Densitometric analysis of proteins on Western blots. Two-way ANOVA test for P-AKT1-S473 versus T-AKT1 optical density ratio followed by Bonferroni's multiple comparisons was employed to analyze the data. The results of [time (proliferating myoblasts, differentiating myotubes, differentiated myotubes)] amounted to *F*_(2, 42)_ = 67.52, *p* < 0.0001 for ATR; *F*_(2, 42)_ = 9.014, *p* = 0.0006 for SIM; *F*_(2, 24)_ = 3.349, *p* = 0.0521 for M*β*CD. Treatment: ATR, ATR + MEV, ATR + GGOH, ATR + FOH, and ATR + Chol-PEG (*F*_(6, 21)_ = 0.2542, *p* = 0.9520); SIM, SIM + MEV, SIM + GGOH, SIM + FOH, and SIM + Chol-PEG (*F*_(6, 21)_ = 0.2754, *p* = 0.9423); M*β*CD, M*β*CD + MEV, M*β*CD + GGOH, M*β*CD + FOH, and M*β*CD + Chol-PEG (*F*_(5, 12)_ = 0.5688, *p* = 0.7228). Interaction: *F*_(12, 42)_ = 8.344, *p* < 0.0001 for ATR; *F*_(12, 42)_ = 9.014, *p* = 0.0006 for SIM; *F*_(10, 24)_ = 1.055, *p* = 0.42 for M*β*CD. Densitometric analysis of proteins on Western blots. Two-way ANOVA test for P-GSK-3*β*-S9 versus GSK-3*β* optical density ratio followed by Bonferroni's multiple comparisons was employed to analyze the data. The results of [time (proliferating myoblasts, differentiating myotubes, differentiated myotubes)] amounted to *F*_(2, 28)_ = 6.199, *p* = 0.0059 for ATR; *F*_(2, 28)_ = 65.48, *p* < 0.0001 for SIM; and *F*_(1, 12)_ = 22.25, *p* < 0.0001 for M*β*CD. Treatment: ATR, ATR + MEV, ATR + GGOH, ATR + FOH, and ATR + Chol-PEG (*F*_(6, 14)_ = 41.29, *p* < 0.0001); SIM, SIM + MEV, SIM + GGOH, SIM + FOH, and SIM + Chol-PEG (*F*_(6, 14)_ = 0.6259, *p* = 0.7074); M*β*CD, M*β*CD + MEV, M*β*CD + GGOH, M*β*CD + FOH, and M*β*CD + Chol-PEG (*F*_(5, 6)_ = 0.1876, *p* = 0.9568). Interaction: *F*_(12, 28)_ = 3.455, *p* = 0.0033 for ATR; *F*_(12, 28)_ = 2.057, *p* = 0.0568 for SIM; *F*_(10, 12)_ = 0.8854, *p* = 0.5705 for M*β*CD. GGOH protected proliferating myoblasts and differentiating myotubes but not differentiated myotubes from the cytotoxic effects of statins and M*β*CD which are evidenced by elevated P-AKT1-S473 and P-GSK-3*β*-S9 (Figures 3(a) and 3(b)). Similarly to GGOH, FOH, and Chol-PEG, P-AKT1-S473 and P-GSK-3*β*-S9 protein expression levels in differentiating myotubes were increased but not in proliferating myoblasts or differentiated myotubes.

### 3.4. GGOH Reversal of Statin-Dependent Decrease in Muscle Cell Viability Is Associated with Increased Prenylation of the RAP GTPase

**Figure 4:**
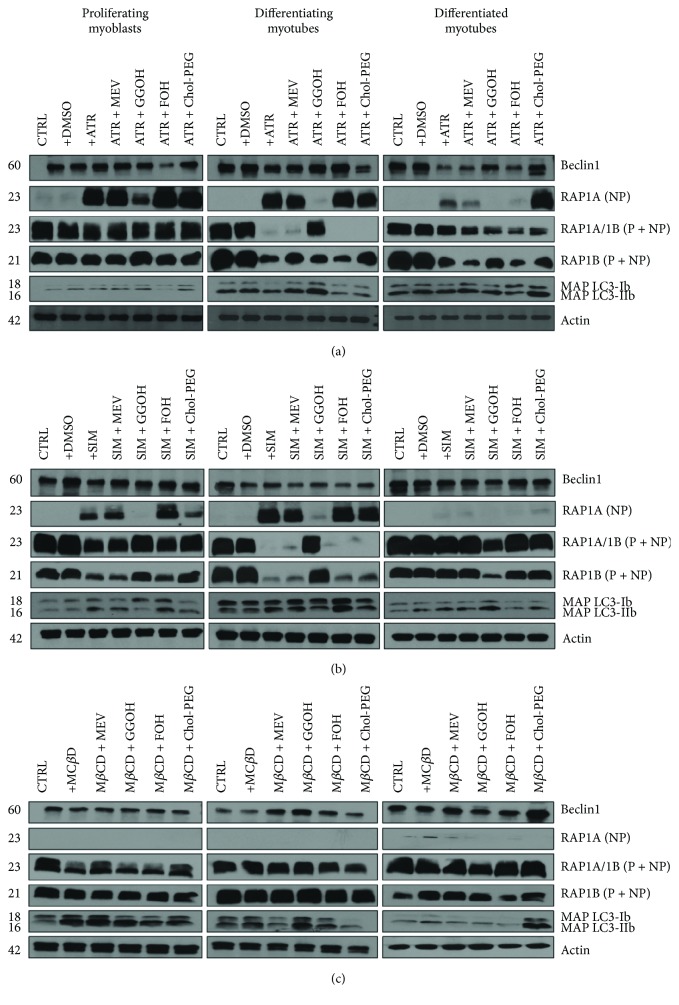
Effect of nonsterol isoprenoids and soluble cholesterol treatments on Beclin 1 and MAP LC3-Ib/MAP LC3-IIb and RAP GTPase in C2C12 myoblasts affected by statins or M*β*CD.

In addition to decreasing HMG-CoA reductase activity, statins also reduce the synthesis of isoprenoid side products of cholesterol synthesis, such as geranylgeranyl pyrophosphate (GGPP) and farnesyl pyrophosphate (FPP) [3]. In the skeletal muscle, RAP GTPase is known to be exclusively prenylated by GGOH [28]; thus, it was of interest to determine the nonprenylated and prenylated RAP1A/B protein expression levels. In nontreated control cells, nonprenylated RAP1A was not detected (Figure 4(a)). Total protein was extracted from differentiating C2C12 myoblasts exposed for 24, 72, or 120 h to statins or M*β*CD (IC_50_) (day 1—proliferating myoblasts, day 3—differentiating myotubes, and day 5—differentiated myotubes). ATR or SIM was administered in the listed concentration at each day of differentiation: ATR: proliferating myoblasts—76 *μ*M, differentiating myotubes—46 *μ*M, and differentiated myotubes—36 *μ*M; SIM: proliferating myoblasts—87 *μ*M, proliferating myoblasts—6 *μ*M, and proliferating myoblasts—3 *μ*M or vehicle control (0.1% DMSO or 2% HS DMEM) without or with selected mevalonate pathway intermediate (mevalonate 100 *μ*M, geranylgeraniol 10 *μ*M, farnesol 10 *μ*M, and Chol-PEG 1 mM). (a) Atorvastatin (ATR, IC_50_) inhibited the Beclin 1 protein expression levels in differentiated myotubes irrespective to cotreatment except the Chol-PEG (1 mM) cotreatment which reversed statin-dependent effect during in vitro myogenesis. On the contrary, Chol-PEG further lessens Beclin 1 protein level reduced by now in ATR-treated differentiating myotubes. In turn, FOH (10 *μ*M) was the sole factor which diminished Beclin 1 in proliferating myoblasts. ATR (IC_50_) significantly reduced the amount of MAP LC3-I/IIb compared to untreated cells in differentiating myotubes. MEV overturned ATR effect, whereas GGOH markedly elevated the MAP LC3-IIb expression level even above the untreated control. Neither ATR nor isoprenoids could affect MAP LC3-I/IIb protein expression levels in proliferating myoblasts and differentiated myotubes. SIM does not seem to be as effective as ATR in MAP LC3-I/IIb processing. (b) For comparison, SIM-dependent effect on Beclin 1 was apparently more accentuated in differentiating myotubes. In contrast to ATR, however, GGOH protected SIM-dependent decline of MAP LC3-IIb in differentiated myotubes (Figure 3(b)). (c) M*β*CD had no visible effect during myogenesis; however, MEV (100 *μ*M) and GGOH (10 *μ*M) cotreatment increased Beclin 1 levels in differentiating myotubes whereas Chol-PEG (1 mM) in differentiated myotubes. M*β*CD as cholesterol chelator increased MAP LC3-IIb in proliferating myoblasts, and none of the cotreatment affected this effect. Chol-PEG significantly elevated MAP LC3-I/IIb expression levels in differentiated myotubes. Representative blots are shown in (a), (b), and (c) from three independent repeats. Densitometric analysis of proteins on Western blots. Two-way ANOVA test for Beclin 1 versus actin optical density ratio followed by Bonferroni's multiple comparisons was employed to analyze the data. The results of [time (proliferating myoblasts, differentiating myotubes, differentiated myotubes)] amounted to *F*_(2, 28)_ = 34.73, *p* < 0.0001 for ATR; *F*_(2, 28)_ = 30.22, *p* < 0.0001 for SIM; and *F*_(2, 24)_ = 2.012, *p* = 0.1557 for M*β*CD. Treatment: ATR, ATR + MEV, ATR + GGOH, ATR + FOH, and ATR + Chol-PEG (*F*_(6, 14)_ = 0.3566, *p* = 0.8943); SIM, SIM + MEV, SIM + GGOH, SIM + FOH, and SIM + Chol-PEG (*F*_(6, 14)_ = 30.22, *p* = 0.0822); M*β*CD, M*β*CD + MEV, M*β*CD + GGOH, M*β*CD + FOH, and M*β*CD + Chol-PEG (*F*_(5, 12)_ = 0.2727, *p* = 0.0229). Interaction *F*_(12, 28)_ = 1.139, *p* = 0.3707 for ATR; *F*_(12, 28)_ = 2.168, *p* = 0.0845 for SIM; *F*_(10, 24)_ = 0.2727, *p* = 0.9194 for M*β*CD. Densitometric analysis of proteins on Western blots. Two-way ANOVA test for MA PLC-II3b to MAP LC3-Ib optical density ratio followed by Bonferroni's multiple comparisons was employed to analyze the data. The results of [time (proliferating myoblasts, differentiating myotubes, differentiated myotubes)] amounted to *F*_(2, 28)_ = 1.135, *p* = 0.3358 for ATR; *F*_(2, 28)_ = 19.56, *p* < 0.0001 for SIM; and *F*_(2, 24)_ = 12.30, *p* = 0.0002 for M*β*CD. Treatment: ATR, ATR + MEV, ATR + GGOH, ATR + FOH, and ATR + Chol-PEG (*F*_(6, 14)_ = 1.782, *p* = 0.1748); SIM, SIM + MEV, SIM + GGOH, SIM + FOH, and SIM + Chol-PEG (*F*_(6, 14)_ = 4.149, *p* = 0.0422); M*β*CD, M*β*CD + MEV, M*β*CD + GGOH, M*β*CD + FOH, and M*β*CD + Chol-PEG (*F*_(5, 12)_ = 3.995, *p* = 0.0229). Interaction *F*_(12, 28)_ = 1.139, *p* = 0.3707 for ATR; *F*_(12, 28)_ = 2.168, *p* = 0.0845 for SIM; *F*_(10, 24)_ = 0.6263, *p* = 0.777 for M*β*CD. Densitometric analysis of proteins on Western blots. Two-way ANOVA test for RAP1A (NP) versus actin optical density ratio followed by Bonferroni's multiple comparisons was employed to analyze the data. The results of [time (proliferating myoblasts, differentiating myotubes, differentiated myotubes)] amounted to *F*_(2, 28)_ = 69.12, *p* < 0.0001 for ATR; *F*_(2, 28)_ = 17.89, *p* < 0.0001 for SIM; and *F*_(2, 12)_ = 8.616, *p* = 0.0048 for M*β*CD. Treatment: ATR, ATR + MEV, ATR + GGOH, ATR + FOH, and ATR + Chol-PEG (*F*_(6, 14)_ = 32.63, *p* < 0.0001); SIM, SIM + MEV, SIM + GGOH, SIM + FOH, and SIM + Chol-PEG (*F*_(6, 14)_ = 28.30, *p* < 0.0001); M*β*CD, M*β*CD + MEV, M*β*CD + GGOH, M*β*CD + FOH, and M*β*CD + Chol-PEG (*F*_(5, 6)_ = 0.3290, *p* = 0.8785). Interaction: *F*_(12, 28)_ = 5.224, *p* = 0.0002 for ATR; *F*_(12, 28)_ = 2.708, *p* = 0.0146 for SIM; *F*_(10, 24)_ = 1.178, *p* = 0.3885 for M*β*CD. Densitometric analysis of proteins on Western blots. Two-way ANOVA test for RAP1B (P + NP) versus actin optical density ratio followed by Bonferroni's multiple comparisons was employed to analyze the data. The results of [time (proliferating myoblasts, differentiating myotubes, differentiated myotubes)] amounted to *F*_(2, 28)_ = 11.00, *p* = 0.0003 for ATR; *F*_(2, 28)_ = 11.43, *p* = 0.0002 for SIM; and *F*_(2, 24)_ = 1.077, *p* = 0.3567 for M*β*CD. Treatment: ATR, ATR + MEV, ATR + GGOH, ATR + FOH, and ATR + Chol-PEG (*F*_(6, 14)_ = 0.9608, *p* = 0.4852); SIM, SIM + MEV, SIM + GGOH, SIM + FOH, and SIM + Chol-PEG (*F*_(6, 14)_ = 1.042, *p* < 0.4397); M*β*CD, M*β*CD + MEV, M*β*CD + GGOH, M*β*CD + FOH, and M*β*CD + Chol-PEG (*F*_(5, 12)_ = 0.1311, *p* = 0.9822). Interaction: *F*_(12, 28)_ = 1.604, *p* = 0.1472 for ATR; *F*_(12, 28)_ = 1.397, *p* = 0.2251 for SIM; *F*_(2, 24)_ = 0.3767, *p* = 0.9449 for M*β*CD. Densitometric analysis of proteins on Western blots. Two-way ANOVA test for RAP1A/RAP1B (P + NP) versus actin optical density ratio followed by Bonferroni's multiple comparisons was employed to analyze the data. The results of [time (proliferating myoblasts, differentiating myotubes, differentiated myotubes)] amounted to *F*_(2, 14)_ = 715.96, *p* < 0.0001 for ATR; *F*_(2, 14)_ = 23.47, *p* < 0.0001 for SIM; and *F*_(2, 12)_ = 15.75, *p* = 0.0004 for M*β*CD. Treatment: ATR, ATR + MEV, ATR + GGOH, ATR + FOH, and ATR + Chol-PEG (*F*_(6, 7)_ = 29.38, *p* < 0.0001); SIM, SIM + MEV, SIM + GGOH, SIM + FOH, and SIM + Chol-PEG (*F*_(6, 7)_ = 1.41, *p* = 0.3286); M*β*CD, M*β*CD + MEV, M*β*CD + GGOH, M*β*CD + FOH, and M*β*CD + Chol-PEG (*F*_(5, 6)_ = 0.09, *p* = 0.9918). Interaction: *F*_(12, 14)_ = 41.07, *p* < 0.0001 for ATR; *F*_(12, 14)_ = 2.08, *p* = 0.0968 for SIM; *F*_(10, 12)_ = 0.76, *p* = 0.6644 for M*β*CD. ATR treatment increased nonprenylated RAP1A GTPase protein expression levels throughout the myogenesis (Figure 4(a)). This effect was also observed in ATR treatments combined with addition of MEV, FOH, and Chol-PEG but not with incorporation of GGOH. Evidently, GGOH reversed ATR effect by increasing the amount of prenylated RAP1A in proliferating myoblasts and differentiating myotubes (Figure 4(a)). Chol-PEG increased nonprenylated RAP1A but neither elevated prenylated RAP1B nor RAP1A/1B protein expression levels in day 5 ATR-treated myoblasts. Comparable data were obtained from SIM-treated muscle cells, with the exception of a very weak response obtained in the case of nonprenylated RAP1A and GGOH action in differentiated myotubes (Figure 4(b)). Although GGOH strongly stimulated RAP1 prenylation—evaluated by RAP1A/1B and RAP1B protein expression levels—in proliferating myoblasts and differentiating myotubes, the compound exhibited an opposite effect in SIM-treated differentiated myotubes (Figure 4(b)).

### 3.5. Isoprenoids Differentially Affect the Autophagy Markers Beclin 1 and MAP LC3-I/IIb in Muscle Cells Subjected to Statin and M*β*CD Treatment

Beclin 1 as a vital component for omegasome formation is an important element for the induction of autophagy acting as a cellular switch between apoptosis and autophagy [29]. IC_50_ concentrations of ATR inhibited expression levels of Beclin 1 protein in differentiated myotubes irrespective to the cotreatment agent with the exception of Chol-PEG which completely reversed the statin-dependent effect (Figure 4(a)). Notably, Chol-PEG was less effective in restoring Beclin 1 protein levels in ATR-treated differentiating myotubes. In ATR-treated proliferating myoblasts, only FOH was able to decrease Beclin 1 expression. The SIM-dependent effect on Beclin 1 appeared more accentuated in differentiating myotubes. M*β*CD had no visible effect during myogenesis, albeit cotreatment with MEV, and GGOH increased Beclin 1 levels in differentiating myotubes, whereas Chol-PEG was active in differentiated myotubes (Figure 4(b)).

MAP LC3-I/IIb processing is a fundamental step in phagophore organization [30]. To be embedded within the phagophore double membrane, MAP LC3-Ib must be first lipidated to MAP LC3-IIb. Consequently, increased ratio of MAP LC3-IIb to MAP LC3-Ib protein expression is an indication of autophagy initiation [29]. ATR significantly reduced the MAP LC3-IIb/Ib with regard to untreated cells in differentiating myotubes (Figure 4(a)), an effect reversed by MEV and GGOH. In difference, GGOH markedly elevated MAP LC3-IIb expression even above the levels in untreated controls (Figure 4(a)). Neither ATR nor isoprenoids changed MAP LC3-IIb/Ib expression in proliferating myoblasts and differentiated myotubes. SIM does not appear as effective as ATR in MAP LC3-Ib to MAP LC3-IIb processing. Similarly to ATR, GGOH protected SIM-dependent loss of MAP LC3-IIb in differentiated myotubes (Figure 4(b)). M*β*CD as a cholesterol chelator increased MAP LC3-IIb in proliferating myoblasts and none of the cotreatments rescued this effect (Figure 4(c)). In contrast, Chol-PEG significantly elevated MAP LC3-IIb/Ib expression in M*β*CD-treated differentiated myotubes (Figure 4(c)). Overall, these data suggest that statins have the capacity to delay autophagy by weakening MAP LC3-Ib lipidation to MAP LC3-IIb and that the SIM-dependent effect on MAP LC3-Ib to MAP LC3-IIb processing was delayed. GGOH was the most potent compound tested herein capable of restoring cell viability through cytoprotective autophagy.

### 3.6. Changes in Autophagy Activation Are Opposite in Pattern to Apoptotic Index

**Figure 5:**
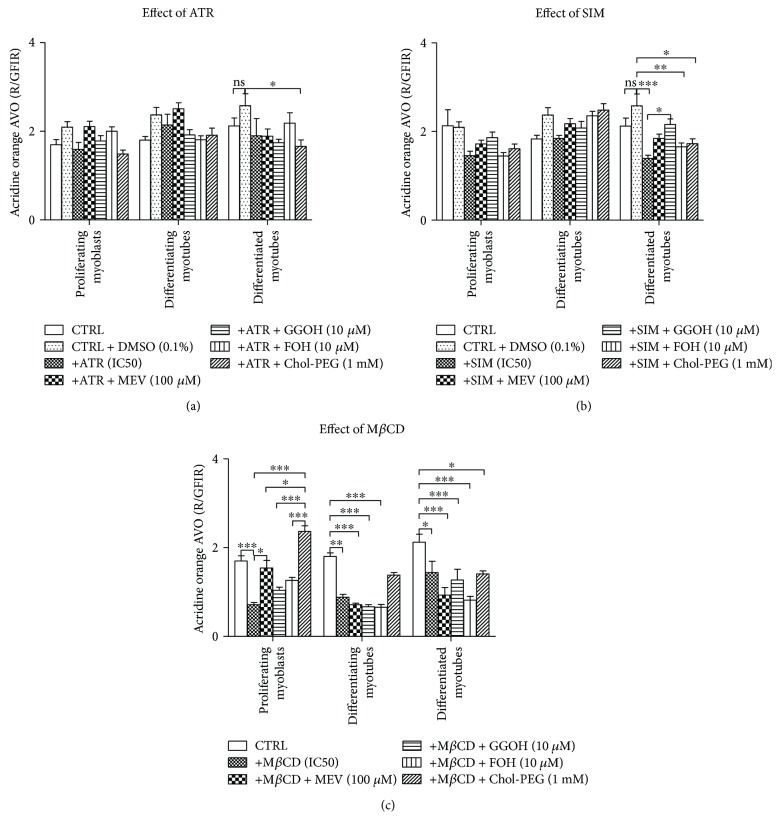
Effect of nonsterol isoprenoids and soluble cholesterol treatments on acidic vacuolar organelles (AVO) as red to green fluorescence intensity ratio (R/GFIR) in C2C12 myoblasts affected by statins or M*β*CD. C2C12 myoblasts were exposed for 24, 72, or 120 h to statins or M*β*CD (IC_50_) (day 1—proliferating myoblasts, day 3—differentiating myotubes, and day 5—differentiated myotubes). ATR or SIM was administered in the listed concentration at each day of differentiation: ATR: day 1—76 *μ*M, day 3—46 *μ*M, and day 5—36 *μ*M; SIM: day 1—87 *μ*M, day 3—6 *μ*M, and day 5–3 *μ*M. M*β*CD was added to give the final concentration: day 1—2.7 mM, day 3—1.9 mM, and day 5—1.1 mM or vehicle control (0.1% DMSO or 2% HS DMEM) without or with selected mevalonate pathway intermediate (mevalonate 100 *μ*M, geranylgeraniol 10 *μ*M, farnesol 10 *μ*M, Chol-PEG 1 mM, dolichol 1 *μ*g/mL, and ubiquinol 10 *μ*g/mL). Next, cells were subjected to vital staining with acridine orange (see Materials and Methods). Red to green fluorescence intensity ratio (R/GFIR) was calculated in at least 10 replicates for each treatment and nontreated controls. Two-way ANOVA test for R/GFIR followed by Bonferroni's multiple comparisons was employed to analyze the data. The results of [time (proliferating myoblasts, differentiating myotubes, differentiated myotubes)] amounted to *F*_(2,191)_ = 3.774, *p* = 0.0247 for ATR; *F*_(2,194)_ = 11.21, *p* < 0.0001 for SIM; and *F*_(2,189)_ = 11.78, *p* < 0.0001 for M*β*CD. Treatment: ATR, ATR + MEV, ATR + GGOH, ATR + FOH, and ATR + Chol-PEG (*F*_(6,191)_ = 5.084, *p* < 0.0001); SIM, SIM + MEV, SIM + GGOH, SIM + FOH, and SIM + Chol-PEG (*F*_(6,194)_ = 6.814, *p* < 0.0001); M*β*CD, M*β*CD + MEV, M*β*CD + GGOH, M*β*CD + FOH, and M*β*CD + Chol-PEG (*F*_(5,189)_ = 23.42, *p* < 0.0001). Interaction: *F*_(12,191)_ = 1.450, *p* = 0.1464 for ATR; *F*_(12,194)_ = 2.604, *p* = 0.0031 for SIM; *F*_(10,189)_ = 5.532, *p* < 0.0001 for M*β*CD. Error bars = SEM and ^∗^*p* < 0.05, ^∗∗^*p* < 0.01, and ^∗∗∗^*p* < 0.001 for comparison between the means. Results are means of three independent experiments.

Evaluation of acidic vacuolar organelles (AVO) with AO to assess autophagy revealed that ATR either alone or together with MEV, GGOH, FOH, or Chol-PEG did not affect AVO formation (Supplementary data 3). In difference to proliferating myoblasts and differentiating myotubes, AVO formation of myotubes at day 5 was significantly reduced by ATR alone or in cotreatment (Figure 5(a)). Similarly, SIM treatment limited AVO number only at day 5, but in this case, GGOH successfully reversed this effect (Figure 5(b)). The most negative effect on AVO representation was evoked by M*β*CD (Figure 5(c)). M*β*CD significantly reduced AVO at days 1, 3, and 5 of differentiation. Indeed, the only metabolite capable of reversing this effect was Chol-PEG which was active at days 1 and 3 but could hardly recover AVO at day 5.

### 3.7. Prenylation with GGOH Is Critical for Maintaining Statin-Repressed Muscle Cell Viability

**Figure 6:**
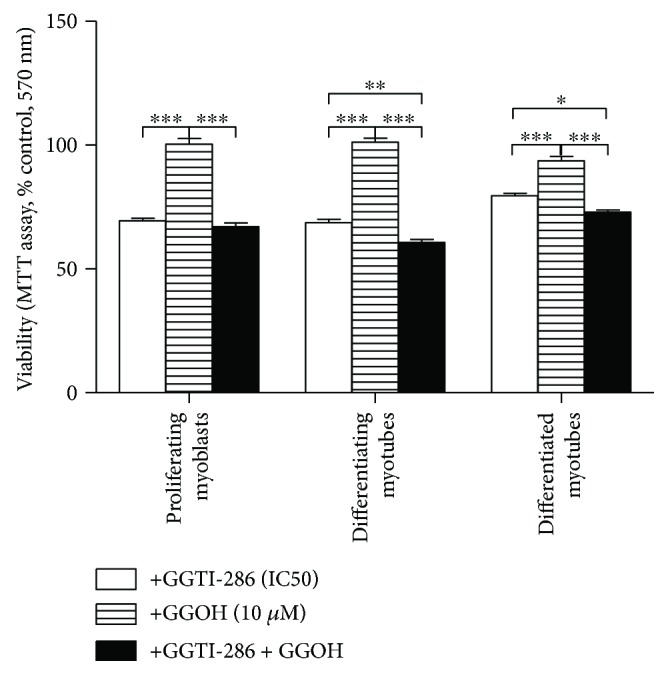
Geranylgeraniol (GGOH) does not rescue C2C12 myoblasts from GGTI-286-induced (IC_50_) compromised cell viability. GGTI-286 was used as a specific inhibitor of protein geranylgeranyltransferases. C2C12 myoblasts were exposed for 24, 72, or 120 h to GGTI-286 (day 1—proliferating myoblasts, day 3—differentiating myotubes, and day 5—differentiated myotubes). GGTI-286 (IC_50_): day 1—25 *μ*M, day 3—24 *μ*M, and day 5—23 *μ*M or vehicle control (0.1% DMSO or 2% HS DMEM) without or with GGOH. GGOH (10 *μ*M) could not reverse the drop in cell viability induced by GGTI-286. Two-way ANOVA test [time (proliferating myoblasts, differentiating myotubes, differentiated myotubes)] amounted to *F*_(2, 42)_ = 10.69, *p* = 0.0002; treatment: GGTI-286, GGOH, and GGTI-286 + GGOH: *F*_(2, 21)_ = 346.8, *p* < 0.0001; interaction: *F*_(4, 42)_ = 18.73, *p* < 0.0001, followed by Bonferroni's multiple comparisons employed to analyze the data. Error bars = SEM and ^∗^*p* < 0.05, ^∗∗^*p* < 0.01, and ^∗∗∗^*p* < 0.001 for comparison between the means. Results are means of three independent experiments.

An inhibitor of the protein geranylgeranyltransferases, GGTI-286, was administered at half-maximal cell viability inhibitory concentration (IC_50_) at days 1, 3, and 5 of myogenesis. While separate treatment with GGOH did not affect cell viability, addition of GGTI-286, alone or in combination with GGOH, significantly changed MTT reduction levels highlighting a fundamental role for geranylgeranyltransferases but not GGOH itself (Figure 6). The addition of GGOH to GGTI-286-treated cultures even diminished cell viability compared to that to GGTI-286-treated cells in differentiating and differentiated myotubes (Figure 6, *p* < 0.05). Overall, these data point to protein prenylation as a possible mechanism for GGOH protection against statin-induced impairment of myoblast viability during myogenesis and suggest that GGOH becomes harmful when geranylgeranyltransferases are withdrawn from the prenylation process.

### 3.8. Effect of Isoprenoids on Statin- and M*β*CD-Mediated Blocked Myogenesis

**Figure 7:**
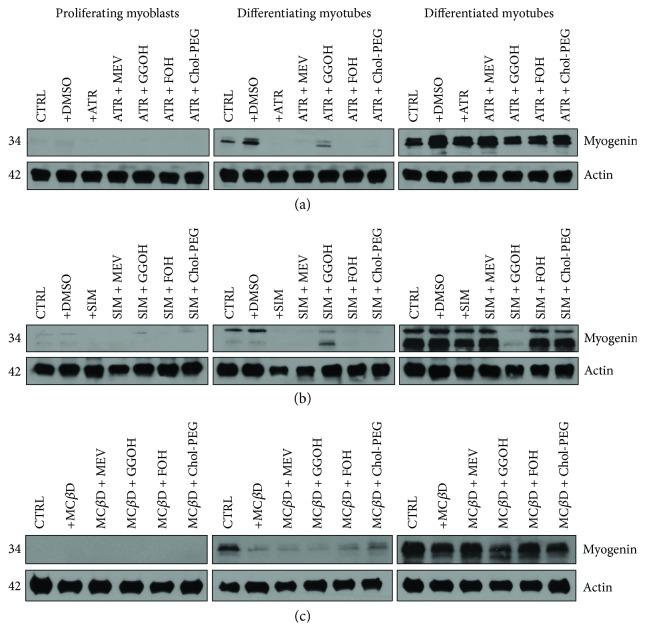
Effect of nonsterol isoprenoids and soluble cholesterol treatments on myogenin in C2C12 myoblasts affected by statins or M*β*CD.

Myogenin transcription factor is a widely known specific marker of terminal differentiation of muscle cells undergoing fusion to form cell syncytium, a multinucleated myotube resulting from multiple fusions of mononuclear myoblasts. As anticipated, no myogenin was observed in proliferating myoblasts (Figure 7). Total protein was extracted from differentiating C2C12 myoblasts exposed for 24, 72, or 120 h statins or M*β*CD (IC_50_) (day 1—proliferating myoblasts, day 3—differentiating myotubes, and day 5—differentiated myotubes). ATR or SIM was administered in the listed concentration at each day of differentiation: ATR: day 1—76 *μ*M, day 3—46 *μ*M, and day 5—36 *μ*M; SIM: day 1—87 *μ*M, day 3—6 *μ*M, and day 5—3 *μ*M. M*β*CD was added to give the final concentration: day 1—2.7 mM, day 3—1.9 mM, and day 5—1.1 mM or vehicle control (0.1% DMSO or 2% HS DMEM) without or with selected mevalonate pathway intermediate (mevalonate 100 *μ*M, geranylgeraniol 10 *μ*M, farnesol 10 *μ*M, and Chol-PEG 1 mM). No myogenin expression was detected at day 1 regardless of treatment. (a) Atorvastatin (ATR, IC_50_) inhibited myogenin expression level at day 3 but not at day 5. Only GGOH (10 *μ*M) partially reversed the ATR effect by accelerating myogenin amount at day 3. In turn, at day 5, GGOH cotreatment caused moderate fall in myogenin expression. (b) Correspondingly, simvastatin (SIM, IC_50_) decreased myogenin expression levels at day 3 without any noticeable effect at day 5. Similarly to ATR, addition of GGOH (10 *μ*M) reduced myogenin expression level at day 5. (c) Methyl-beta-cyclodextrin (M*β*CD, IC_50_) markedly reduced myogenin amount at day 3, even though it could hardly affect the expression levels at day 5. A representative blots are shown in (a), (b), and (c) from three independent repeats. Densitometric analysis of proteins on Western blots. Two-way ANOVA test for myogenin versus actin optical density ratio followed by Bonferroni's multiple comparisons was employed to analyze the data. The results of [time (proliferating myoblasts, differentiating myotubes, differentiated myotubes)] amounted to *F*_(2, 28)_ = 6.444, *p* = 0.0050 for ATR; *F*_(2, 28)_ = 86.45, *p* < 0.0001 for SIM; and *F*_(2, 24)_ = 127.7, *p* < 0.0001 for M*β*CD. Treatment: ATR, ATR + MEV, ATR + GGOH, ATR + FOH, and ATR + Chol-PEG (*F*_(6, 14)_ = 0.8277, *p* = 0.5675); SIM, SIM + MEV, SIM + GGOH, SIM + FOH, and SIM + Chol-PEG (*F*_(6, 14)_ = 1.385, *p* = 0.2870); M*β*CD, M*β*CD + MEV, M*β*CD + GGOH, M*β*CD + FOH, and M*β*CD + Chol-PEG (*F*_(5, 12)_ = 3.212, *p* = 0.0453. Interaction: *F*_(12, 28)_ = 0.1894, *p* = 0.9972 for ATR; *F*_(12, 28)_ = 4.333, *p* = 0.0007 for SIM; *F*_(10, 24)_ = 4.305, *p* = 0.0016 for M*β*CD. ATR, SIM, and M*β*CD at their respective (IC_50_) concentrations significantly reduced myogenin levels in day 3 and 5 differentiating myotubes (Figures 7(a) and 7(b), resp.). Notably, cotreatment of ATR and SIM with GGOH reversed the statin-dependent inhibition of myogenin expression. No effect was observed by cotreatment with either mevalonate intermediate or Chol-PEG on M*β*CD-dependent loss of myogenin amounts in differentiating myotubes (Figure 7(c)).

### 3.9. Lack of Effect of Statins, M*β*CD, and Isoprenoids on Expression Levels of Myosin Heavy Chain during Myogenesis

**Figure 8:**
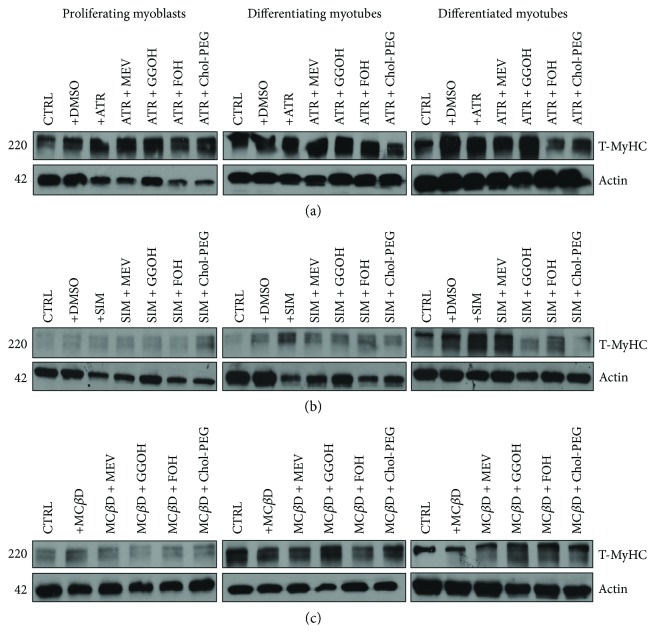
Effect of nonsterol isoprenoids and soluble cholesterol treatments on total myosin heavy chain (T-MyHC) amount in C2C12 myoblasts affected by statins or M*β*CD.

Myosin heavy chain protein is a representative product of the myogenin-mediated activation of the *MyHC* target gene (Supplementary data 4), and therefore, its expression levels during myogenesis are expected to correlate with changes in myogenin expression. Surprisingly, MyHC protein expression levels were hardly affected by statins or M*β*CD and neither isoprenoids nor Chol-PEG had any effect on its expression levels throughout myogenesis (Figure 8). Total protein was extracted from differentiating C2C12 myoblasts exposed for 24, 72, or 120 h to statin (IC_50_) or M*β*CD (IC_50_) (day 1—proliferating myoblasts, day 3—differentiating myotubes, and day 5—differentiated myotubes). ATR or SIM was administered in the listed concentration at each day of differentiation: ATR: day 1—76 *μ*M, day 3—46 *μ*M, and day 5—36 *μ*M; SIM: day 1—87 *μ*M, day 3—6 *μ*M, and day 5—3 *μ*M. M*β*CD was added to give the final concentration: day 1—2.7 mM, day 3—1.9 mM, and day 5—1.1 mM or vehicle control (0.1% DMSO or 2% HS DMEM) without or with selected mevalonate pathway intermediate (mevalonate 100 *μ*M, geranylgeraniol 10 *μ*M, farnesol 10 *μ*M, and Chol-PEG 1 mM). (a) Atorvastatin (ATR, IC_50_) did not affect MyHC protein expression levels during in vitro myogenesis. Eventually, mevalonate (MEV, 100 *μ*M), farnesol (FOH, 10 *μ*M), and Chol-PEG (1 mM) cotreatment significantly elevated MyHC protein at day 1. (b) Simvastatin (SIM, IC_50_) did not change MyHC protein levels irrespective to time and type of treatment. (c) Also, methyl-beta-cyclodextrin (M*β*CD, IC_50_) could not effect MyHC, but in contrast to ATR and SIM, cotreatment with GGOH (10 mM) augmented MyHC expression levels at days 3 and 5. Representative blots are shown in (a), (b), and (c) from three independent repeats. Densitometric analysis of proteins on Western blots. Two-way ANOVA test for MyHC versus actin optical density ratio followed by Bonferroni's multiple comparisons was employed to analyze the data. The results of [time (proliferating myoblasts, differentiating myotubes, differentiated myotubes)] amounted to *F*_(2, 42)_ = 108.6, *p* < 0.0001 for ATR; *F*_(2, 42)_ = 1.707, *p* = 0.1997 for SIM; and *F*_(2, 12)_ = 155.9, *p* < 0.0001 for M*β*CD. Treatment: ATR, ATR + MEV, ATR + GGOH, ATR + FOH, and ATR + Chol-PEG (*F*_(6, 21)_ = 12.76, *p* < 0.0001); SIM, SIM + MEV, SIM + GGOH, SIM + FOH, and SIM + Chol-PEG (*F*_(6, 14)_ = 2.149, *p* < 0.1118); M*β*CD, M*β*CD + MEV, M*β*CD + GGOH, M*β*CD + FOH, and M*β*CD + Chol-PEG (*F*_(5, 6)_ = 6.297, *p* = 0.0222). Interaction *F*_(12, 42)_ = 10.51, *p* < 0.0001 for ATR; *F*_(12, 28)_ = 1.163, *p* = 0.3546 for SIM; *F*_(10, 12)_ = 8.718, *p* = 0.0004 for M*β*CD.

### 3.10. Statins and M*β*CD Negatively Influence Myotube Index at Later Stages of Myogenesis, an Effect Resistant to Nonsterol Isoprenoids and Cholesterol

**Figure 9:**
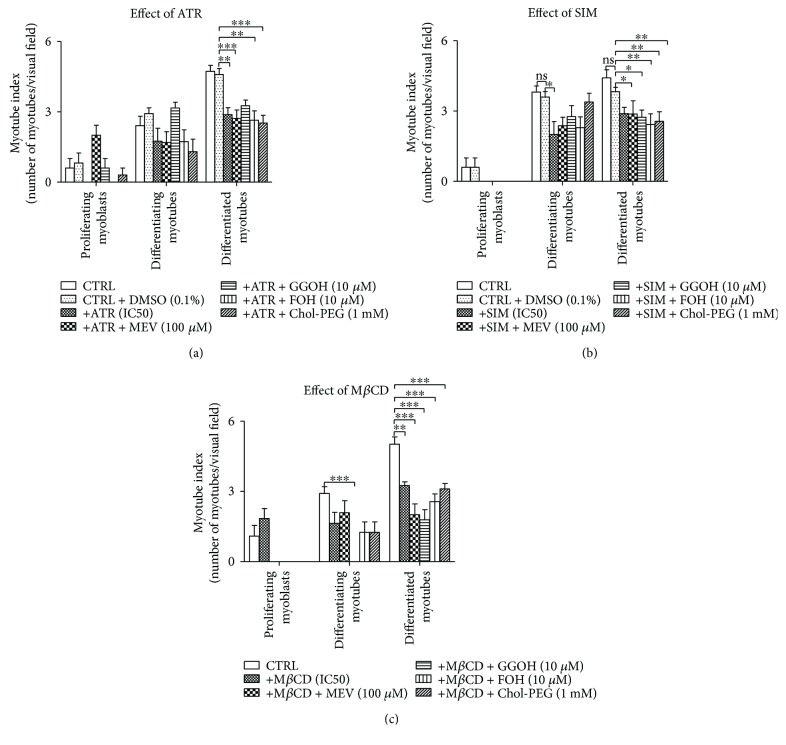
Effect of nonsterol isoprenoids and soluble cholesterol treatments on myotube index (MI) in C2C12 myoblasts affected by statins or M*β*CD. C2C12 myoblasts were exposed for 24, 72, or 120 h to statins or M*β*CD (IC_50_) (day 1—proliferating myoblasts, day 3—differentiating myotubes, and day 5—differentiated myotubes). ATR or SIM was administered in the listed concentration at each day of differentiation: ATR: day 1—76 *μ*M, day 3—46 *μ*M, and day 5—36 *μ*M; SIM: day 1—87 *μ*M, day 3—6 *μ*M, and day 5—3 *μ*M. M*β*CD was added to give the final concentration: day 1—2.7 mM, day 3—1.9 mM, and day 5–1.1 mM or vehicle control (0.1% DMSO or 2% HS DMEM) without or with selected mevalonate pathway intermediate (mevalonate 100 *μ*M, geranylgeraniol 10 *μ*M, farnesol 10 *μ*M, Chol-PEG 1 mM, dolichol 1 *μ*g/mL, and ubiquinol 10 *μ*g/mL). Next, cells were subjected to immunofluorescence with HO33342 to counterstain nuclei (see Materials and Methods). The myotube index was determined as the ratio of the nuclear number in myotubes (C2C12 cells with three or more nuclei) to the total number of nuclei multiplied by 100%). (a) Atorvastatin (ATR, IC_50_) did not affect myotube index (MI) at days 1 and 3. However, it significantly diminished fraction of myonuclei in myotubes at day 5. Neither of added nonsterol isoprenoids nor cholesterol treatment could influence ATR effect. (b) Simvastatin (SIM, IC_50_) did not change MI at day 1, but it significantly lessened myotube representation at days 3 and 5. Nonsterol isoprenoids and cholesterol reversed SIM effect at day 3 but not at day 5. (c) Methyl-beta-cyclodextrin (M*β*CD, IC_50_) could not effect MI at day 1, but it significantly decreased percentage of myotubes at days 3 and 5 without any effect of nonsterol isoprenoids or cholesterol. Two-way ANOVA test for MI followed by Bonferroni's multiple comparisons was employed to analyze the data. The results of [time (proliferating myoblasts, differentiating myotubes, differentiated myotubes)] amounted to *F*_(2,337)_ = 80.99, *p* < 0.0001 for ATR; *F*_(2,352)_ = 98.91, *p* < 0.0001 for SIM; and *F*_(2,266)_ = 59.00, *p* < 0.0001 for M*β*CD. Treatment: ATR, ATR + MEV, ATR + GGOH, ATR + FOH, and ATR + Chol-PEG (*F*_(6,337)_ = 5.982, *p* < 0.0001); SIM, SIM + MEV, SIM + GGOH, SIM + FOH, and SIM + Chol-PEG (*F*_(6,352)_ = 6.370, *p* < 0.0001); M*β*CD, M*β*CD + MEV, M*β*CD + GGOH, M*β*CD + FOH, and M*β*CD + Chol-PEG (*F*_(5,266)_ = 14.77, *p* < 0.0001). Interaction: *F*_(12,337)_ = 3.096, *p* = 0.0004 for ATR; *F*_(12,352)_ = 0.8962, *p* = 0.5511 for SIM; *F*_(10,266)_ = 2.560, *p* = 0.0057 for M*β*CD. Error bars = SEM and ^∗^*p* < 0.05, ^∗∗^*p* < 0.01, and ^∗∗∗^*p* < 0.001 for comparison between the means. Results are means of three independent experiments.

Both statins (either ATR or SIM at their respective IC_50_ concentrations) and M*β*CD hardly affected myotube index (MI) at day 1 of differentiation (Figure 9). However, SIM and M*β*CD had an effect at day 3, and all of the three compounds—ATR, SIM, and M*β*CD—significantly decreased MI at day 5 (Figures 9(b) and 9(c)). The addition of nonsterol isoprenoids or cholesterol barely influenced this effect.

## 4. Discussion

In the present study, we examined the differential effect of statins (ATR, SIM) and the cholesterol chelator (M*β*CD) in skeletal muscle cells undergoing differentiation process divided into three basic steps: postmitotic proliferation, cell fusion, and myotube enlargement. To this aim, different experimental factors were added (or not) starting at day 1 and continued to day 3 or to day 5 of cell differentiation and the subsequent evaluations were referred to as changes in “proliferating myoblasts,” “differentiating myotubes,” and “differentiated myotubes,” respectively (see Materials and Methods). Upon assessment of the half-maximal cytotoxic concentration (IC_50_), the action of mevalonate pathway intermediates and water-soluble form of cholesterol in cotreatment experiments could clearly be demonstrated. Initially, the nonsterol isoprenoids were selected according to their reliance on mevalonate input blocked by statins (mevalonate (MEV), geranylgeraniol (GGOH), farnesol (FOH), dolichol (DOH), and ubiquinol (UBOH)). Finally, the molecular studies focused on the role of MEV, GGOH, and FOH, compounds that were confronted with water-soluble form of cholesterol, Chol-PEG.

Our data clearly indicate that the decrease in cell viability in proliferating myoblasts caused by statins was significantly reversed by GGOH (Figures 1(a) and 1(b)). On the contrary, Chol-PEG was active in protecting cell viability in proliferating myoblasts and differentiating and differentiated myotubes harmed by M*β*CD (Figure 1(c)). This initial studies demonstrated the particular important role of GGOH in myogenesis in which mevalonate formation is blocked at the level of the HMG-CoA reductase. In turn, in cells severely deprived of cholesterol through administration of M*β*CD, cell viability was restored by Chol-PEG at any step and additionally by DOH in differentiating myotubes, after cell fusion. A different pattern of cell death was found after evaluation of the apoptotic index in statin-treated cells (Figures 2(a) and 2(c)). Neither ATR nor SIM increased the AI which remained unchanged with nonsterol isoprenoids and Chol-PEG cotreatment. Interestingly, a significant increase in AI was observed in differentiating and differentiated myotubes upon M*β*CD treatment (Figure 2(c)) which although significantly reduced by Chol-PEG remained unchanged by nonsterol isoprenoids. These results emphasize the distinct roles played by cholesterol and nonsterol isoprenoids, in particular GGOH, in the detrimental effect on skeletal muscle development exerted by statins and M*β*CD and suggest that statins impair cell viability through GGOH, a vital element for protein prenylation in proliferating myoblasts and differentiating myotubes. A decline in cholesterol had no any significant role in statin-induced myotoxicity as Chol-PEG was unable to restore viability of myocytes affected by statins suggesting that total cholesterol is unlikely participating in statin-mediated myopathy, in agreement with previous findings on cultured muscle cells [16, 28, 31, 32]. Nevertheless, it should be kept in mind that cholesterol controls membrane rigidity and plays a fundamental role in maintaining cell viability at any step of muscle differentiation as demonstrated by M*β*CD-induced myotoxicity (Figures 1(c) and 2(c)).

Several reports have previously described the myotoxic effect of statins [16, 33–35] which was completely prevented by mevalonate and geranylgeraniol and not by farnesol [16]. However, apoptosis induction was only observed in the case of lactone (compound A) or open acid (cerivastatin). Further mechanistic studies have recently revealed that statin lactone-induced myotoxicity takes place as a consequence of the mitochondrial complex III inhibition [36]. The work presented herein, using different elements of the mevalonate and cholesterol pathway to rescue the effect of statins and M*β*CD at different stages of muscle cell differentiation, expands the current knowledge of the molecular mechanisms involved. In contrast to GGOH, MEV—at the concentration tested—failed to protect from ATR- and SIM-induced myotoxicity. Whether the MEV concentration of 100 *μ*M concentration employed in our studies is not sufficient for the required GGOH formation remains to be elucidated. As geranylgeranyltransferase inhibitors cause apoptosis in myotubes and mimic apoptogenic action of lovastatin or cerivastatin [16, 31], it is highly plausible to find a protein candidate that is prenylated by the action of geranylgeranyltransferases and thereby controls muscle viability. RAP1 (Ras-proximate-1) small GTPase is a likely candidate as this GTP-binding protein of the RAS superfamily was reported to accumulate during muscle differentiation [20] where RAP1A and RAP1B subgroup localized to late endosomes/lysosomes [21].

Moving further to characterized the cell signaling mechanisms involved in these interactions, we studied the AKT1 kinase to GSK-3*β* pathway, as this cascade organizes cell survival and has indirect control over the GTPase RAP1, known to be the only small G protein prenylated by GGOH in the skeletal muscle [28]. Providing additional support for our studies, not only has the AKT/mTOR signaling pathway been recently demonstrated to play major role in muscle cell viability [37] but also statins (ATR, SIM) have been shown to inhibit AKT phosphorylation in C2C12 myoblasts [38, 39]. Our immunoblot experiments confirmed the repressive effect not only of ATR and SIM but also of M*β*CD on AKT1 phosphorylation status at serine 473 (P-AKT1 (S473) Figure 3). Correspondingly, there was also a slight reduction in total AKT1 (T-AKT1) protein expression level. Upon additional GGOH administration, AKT1 phosphorylation increased moderately in ATR- and SIM-treated proliferating myoblasts (Figure 3(a)) but not in M*β*CD-challenged myoblasts. Thus, GGOH was able, once more, to reverse—at least in part—the statin-dependent AKT1 dephosphorylation. Changes in GSK-3*β* phosphorylation and protein expression levels followed the pattern observed for AKT1 at every step of myogenesis (the lower the level of T-AKT1 and P-AKT1 (S473), the lower the level of T-GSK-3*β* and P-GSK-3*β* (S9)). The mechanisms by which statins inhibit AKT1 kinase activity remain to be completely elucidated although they seem not to result from a direct action of statins and most likely involve blunted insulin and/or insulin-like growth factor (IGF) signaling due to impaired prenylation and/or *N*-glycosylation of upstream signaling molecules for AKT1 [40]. Insulin and IGF-I receptor require dolichol for processing and membrane translocation, whereas Ras has to be prenylated to act as a messenger. In each of these cases, mevalonate is in shortage as the substrate. Consequently, mevalonate emerges as an indirect cell growth regulator [41]. Since Ras activation requires farnesylation rather than geranylgeranylation and we could scarcely demonstrate an effect of farnesol, we conclude that prenylation of a protein other than Ras was responsible for the GGOH-induced reversal of statin-induced myotoxicity.

Our studies did not monitor AKT/mTOR signaling at the TSC1/TSC2 level. Nevertheless, it is very likely that ATR or SIM inhibited RAP1. To verify the RAP1A contribution to muscle cell viability and cytoprotective autophagy, we studied expression levels of RAP1A/1B together with the autophagy markers Beclin 1 and MAP LC3-Ib/IIb by Western blot analysis. In the past, a few papers described RAP1A as a component of late endocytic compartments in myocytes [20, 21]. Muscle cells are known to activate cytoprotective autophagy in response to a number of injurious factors (starvation, exercise), but there is also suggestion that myogenesis from C2C12 myoblasts is associated with extensive mitophagy [42]. Indeed, the expression levels of Beclin1 were not changed upon ATR or SIM administration albeit they were significantly decreased upon addition of M*β*CD to proliferating myoblasts (Figure 4(a)). In proliferating myoblasts, the amount of Beclin 1 decreased with ATR + FOH treatment, although it raised to control levels in M*β*CD cotreated with Chol-PEG at every step of myogenesis (Figures 4(a)–4(c)). MAP LC3-Ib lipidation to active MAP LC3-IIb form is another sensitive marker of phagophore formation [30]. Consistent with the data presented above, the levels of MAP LC3-IIb with respect to MAP LC3-Ib in ATR- or SIM-treated myocytes did not differ from untreated controls. Nonetheless, Chol-PEG cotreatment caused additional raise in MAP LC3-IIb in M*β*CD-treated proliferating myoblasts and differentiated myotubes, findings that points to a critical role of cholesterol in autophagy.

The present studies combined the visualization of the autophagy process through vital staining of myocytes with the assessment of red fluorescence-emitting acidic vacuolar organelles (AVO) and green fluorescent non-AVO. The calculated R/GFIR index [26] revealed significant decrease of AVO representation in differentiated myotubes challenged with statins (Figures 5(a) and 5(b)). In SIM-treated myocytes, however, GGOH administration significantly raised the AVO representation and restored its level to untreated control cells. The most profound changes in AVO representation were detected upon M*β*CD application (Figure 5(c)). The cholesterol chelator brought R/GFIR index to the lowest levels recorded, but adding Chol-PEG reversed this effect in proliferating myoblasts (*p* < 0.001).

In order to distinguish the differential effects of statins and M*β*CD effects on RAP1 prenylation, the protein expression levels were accomplished in their nonprenylated versus prenylated form. As shown in Figure (4), the untreated control cells did not express the nonprenylated form of RAP1A (NP). A considerable increase in nonprenylated RAP1A (NP) was evident after ATR addition at any step of myogenesis (days 1, 3, and 5) and also at days 1 and 3 after SIM administration (Figures 4(a) and 4(b)). Upon M*β*CD administration, no changes in the amount of RAP1A (NP) were noted (Figure 4(c)). As predicted, nonprenylated form of RAP1A almost disappeared from ATR- and SIM-treated myocytes after GGOH addition. Moreover, GGOH was the only potent nonsterol isoprenoid in reducing RAP1A (NP). Chol-PEG exerted no effect on statin-dependent increase in RAP1A (NP). The amount of prenylated + nonprenylated RAP1A/1B (P + NP) was highest in the untreated control cells and fainted after ATR or SIM administration in differentiating myotubes but not in proliferating myoblasts (Figures 4(a) and 4(b)). To make clear which RAP1 isoform (1A or 1B) was subjected to isoprenylation with GGOH, we also examined RAP1B protein expression levels (P + NP). As can be observed on respective blots, RAP1B (P + NP) represented a great deal of prenylated RAP1 in SIM treated, while RAP1A in ATR-treated myocytes (Figures 4(a) and 4(b)). At any time, adding GGOH to ATR- or SIM-treated muscle cells caused drop in nonprenylated RAP1A. These results clearly demonstrate that GGOH is obviously the prime component for RAP1A/1B prenylation. To conclude whether Geranylgeranyltransferases (GGTs) limit the viability of myocytes through isoprenylation, we performed additional experiment using specific inhibitor GGTI-286. As we assumed, if it is not GGOH itself but isoprenylation that is essential for muscle cell survival, we would not have observed any effect of GGOH in the presence of GGTI-286. The bar chart (Figure 6) provides compelling evidence that unless GGTI-286 is present, GGOH is not able to stimulate viability of myocytes. In addition, GGOH given together with GGTI-286, significantly reduced viability with respect to GGTI-286 alone at day 3 and day 5. The last finding points to somehow striking conclusion that excess GGOH (above GGTs flux) might be detrimental for muscle cells. Analogous suggestion spoken to other organs was suggested in the critical review presented several years ago [4]. It points to the cautious use of statins as drugs and accentuates the need of therapeutic dose as important for inhibiting mevalonate pathway just enough to reduce both the excessive cholesterol and GGOH synthesis. One may ask why is isoprenylation with GGOH so important? Actually, geranylgeranyl pyrophosphate (GGPP) as an intermediate of cholesterol biosynthetic pathway plays a pivotal role as lipid attachment for the posttranslational modification of small GTP-binding proteins including RAP1 [43, 44]. Thus, small GTPases must associate with cellular membranes for activity, and respective membrane attachment is mediated by prenyl (geranylgeranyl) posttranslational modification. Little is known about RAP1 GTPase function in skeletal muscle, even though tuberin, the tuberous sclerosis-2 product (TSC2), possesses specific RAP1GAP activity which represses RAP1 function [14]. Thus, as long as TSC1/TSC2 complex is inhibited by AKT, RAP1 GTPase stimulates mTOR/mLST8/raptor complex and cell growth [13]. Previous work has shown inhibition of muscle differentiation by RAP1A protein as it interacts with various mitogen-activated protein kinase- (MAPK-) activating pathways [45]. Muscle differentiation is not initiated as long as MAPK signaling cascade promotes cell cycle. Accordingly, RAP1 protein might play a role of molecular switch by alternating between an active GTP-bound and an inactive GDP-bound state [46]. In fact, there is strong evidence that RAP1A stimulates osteoblastogenic differentiation through activation of extracellular-regulated protein kinase ERK or p38 MAPK kinase in a Ras-independent manner (noncanonical pathway) [47]. RAP1A might stimulate MAPKs indirectly as it has been reported to interact with B-Raf [48]. Definitely, positive effect of RAP1 prenylation (GGOH) on cell viability was most significant in proliferating myoblasts (Figure 1). After all, elevated muscle cell viability was correlated with the expression levels of prenylated RAP1 (Figure 4).

Our experimental model of myoblast-myotube differentiation is useful to ascertain changes in molecular indices of myogenesis. To do this, we determined MYG and MyHC protein expression levels by immunoblotting. Concurrently, immunocytofluorescent and fluorescent studies were conducted on fixed cells for visualization occurrence of MyHC protein and evaluation of myotube index. As shown on the respective blots (Figure 7), no MYG protein was detected in proliferating myoblasts which is normal in muscle cells prior to withdrawal from the cell cycle. Day 3 of differentiation is critical for *MYG* gene activation, so myogenin protein was noticed in untreated differentiating myotubes but not in statins- or M*β*CD-treated cells except cotreatment with GGOH (Figure 7). Another time, merely GGOH increased in part myogenin levels when given together with ATR or SIM (Figures 7(a) and 7(b)). Such effect was not observed in combined treatment with M*β*CD (Figure 7(c)). After myocyte fusion was completed, neither MEV, FOH, nor Chol-PEG affected MYG expression levels. Surprisingly, a significant decline in myogenin was detected in ATR cotreatment with GGOH or FOH, SIM cotreatment with GGOH, and GGOH cotreatment with M*β*CD (Figure 7). The data suggest that GGOH rescues statin-induced inhibition of myogenin expression during the initial differentiation phase, but this isoprenoid cooperates with statins to block myogenin expression in differentiated myotubes. In contrast to myogenin, MyHC protein expression levels were not significantly altered by ATR, SIM, or M*β*CD given alone or together with nonsterol isoprenoids or Chol-PEG (Figure 8).

The presented data related to myoblast differentiation suggest a dual role played by GGOH. Prenylated RAP1 GTPase (following GGOH addition) limits differentiation by promoting myoblast proliferation, but once cell divisions are completed, the accelerated myogenin expression also induced by prenylated RAP1 promotes cell fusion. This mechanism is consistent with the numerous known biological functions of RAP1 including adhesion, spreading, and migration, all fundamental for muscle syncytium formation [49]. Moreover, the p110 catalytic subunit of PI3-K has been identified as RAP1GTP (active form) effector molecule, and AKT is a well-known PI3-K downstream target [46]. PI3-K/AKT signaling cascade is well recognized for its myogenic activity [50]. As we assume, RAP1 prenylation with GGOH promoted muscle cell proliferation (day 1), but later in postmitotic cells (day 3), RAP1 GTPase accelerated myogenesis leading to faster loss of myogenin protein expression in fully differentiated myotubes (day 5) (Figure 7). Certainly, myocytes treated with ATR and GGOH at day 5 did not differ from nontreated control cells in mean number of myonuclei within myotubes (Figure 9(a)). This result is in marked contrast to the significant fall of MI values upon ATR, SIM, and M*β*CD treatments and cotreatments (Figure 9). Explanatory context of these findings is the pronounced GGOH effect observed in ATR-treated myoblasts.

The most intriguing and fascinating aspect of the current study is the possible link between RAP1 prenylation and autophagy regulation. It is clear from our findings (Supplementary data 3) that numerous AVO observed in muscle cells highlight the importance of cytoprotective autophagy. Noticeable raise in Beclin 1 and MAP LC3-IIb protein expression levels was evident after ATR + GGOH and M*β*CD + GGOH cotreatments in differentiating myotubes (Figures 4(a) and 4(c)). Similarly, MAP LC3-IIb increased upon SIM + GGOH, while MAP LC3-IIb and Beclin 1 in response to M*β*CD + Chol-PEG cotreatment in differentiated myotubes (Figures 4(b) and 4(c)). This could be a blueprint of RAP1-mediated autophagy regulation, although further studies with autophagy flux in the presence and absence of lysosomal or vacuolar degradation inhibitors are needed to confirm amplification of the process.

Statins are highly successful drugs for the prevention of cardiovascular disease. As HMG-CoA inhibitors, the drugs reduce cholesterol synthesis in the liver and indirectly modify circulating cholesterol levels through its elevated hepatic uptake. However, in addition to their effect in cholesterol, statins block the mevalonate pathway responsible for the production of several important molecules including ubiquinone, dolichol, and nonsterol isoprenoids like GGPP and FPP. Eventually, statins may exert cytotoxic effect in skeletal muscles, although the mechanism that underlies this action is not fully understood. Statin-induced depletion of cholesterol does not seem as a likely cause of statin myopathy since inhibition of squalene synthase—also a limiting enzyme in cholesterol synthesis—is not only nonmyotoxic, but may also even be cytoprotective [32, 51]. Therefore, for last three decades, research efforts have been dedicated to explain the causative role of statin-mediated injurious effects in the skeletal muscle. Some reports indicated mitochondria [36, 52–57], Ca^2+^ homeostasis [57, 58], plasma membrane monocarboxylate transporter [59], plasma membrane receptors [40, 41], and ubiquitin ligases [31, 60] as statin prime targets. Recently, new evidence has revealed the importance of impaired geranylgeranylation of proteins as the foundation of statin-associated myopathy [16, 28, 31, 45, 61], a concept contested by work carried out in rhabdomyosarcoma and not regular skeletal muscle cells [8].

The aim of this study was to elucidate the molecular mechanisms of statin-induced myotoxicity as successful strategy to prevent statin-derived side effects is lacking. Statins competitively inhibit HMG-CoA reductase leading to significant decreases in the intracellular synthesis of mevalonate, geranylgeraniol, farnesol, ubiquinone, dolichol, and cholesterol. The current work established the respective half-maximal inhibitory concentrations (IC_50_) and tested ATR and SIM in their ability to reduce HMG-CoA reductase activity in comparison with M*β*CD, a compound that binds and removes cholesterol from plasma membrane. In order to assess which mevalonate pathway intermediate or end product is capable to reverse statin and M*β*CD toxicity, we tested mevalonate (MEV), geranylgeraniol (GGOH), farnesol (FOH), and water-soluble cholesterol (Chol-PEG). Geranylgeraniol, a cell-permeable analogue of geranylgeranyl pyrophosphate (GGPP), reversed statin-induced myotoxicity in proliferating myoblasts and differentiating myotubes but not in M*β*CD-treated myocytes. GGOH-mediated positive effects were entirely dependent on Geranylgeranyltransferases (GGTs) activities. In turn, Chol-PEG restored viability to control levels only in M*β*CD cotreated myocytes. GGOH administration increased expression levels of prenylated RAP1A/1B GTPase. This protein in prenylated form also elevated Beclin1 and MAP LC3-IIb in muscle cells affected by ATR or SIM. Chol-PEG did the same in M*β*CD-treated differentiated myotubes. GGOH had a broader protective effect against statin-induced myotoxicity than MEV, FOH, or Chol-PEG. Most of the GGOH- or Chol-PEG-mediated stimulations of myogenesis were seemingly associated with cytoprotective autophagy. AKT1 and GSK-3*β* kinase phosphorylations increased in parallel to viability promoted by GGOH.

In conclusion, the watchful use of GGOH appears as a useful strategy in restraining myotoxicity of statins without any loss in their anticholesterogenic effect.

## 5. Conclusions

1. Myotoxicity induced by ATR and SIM is associated with the reduced GGOH-dependent prenylation of RAP1 protein.
2. Lower myotoxicity is reflected by the respective increase in AKT 1 (S463) and GSK-3*β* (S9) phosphorylation.
3. Geranylgeranyltransferases (GGTs) control myocyte viability through GGOH, which in excess is likely myotoxic.
4. Cytoprotective autophagy is elevated in myocytes during myogenesis. MEV, GGOH, and Chol-PEG are able to reverse statin- or M*β*CD-impaired autophagy.
5. Plasma membrane cholesterol is fundamental for the survival of M*β*CD-challenged myocytes.
6. Muscle differentiation is impaired by statins or M*β*CD, and nonsterol isoprenoids or Chol-PEG could not reverse this effect.

## Supplementary Materials

Supplementary Materials Supplementary Data 1: establishing the half-maximal inhibitory concentrations (IC_50_). Preliminary experiments were carried out with increasing concentrations of metabolic inhibitors in order to establish IC_50_ concentration (the half-maximal inhibitory concentration IC_50_ is a measure of the effectiveness of a substance in inhibiting a specific biological or biochemical function). A series of concentrations for statins (0–100 *μ*M) were used to determine cell viability in the subsequent days of myogenesis (day 1—proliferating myoblasts, day 3—differentiating myotubes, and day 5—differentiated myotubes). Dose-response curves represent least squares of nonlinear Gaussian distribution. The IC_50_ values of factor for each day (1, 3, and 5) were found by extrapolation 50% cell viability (red dotted line) to the horizontal coordinate (abscissa). Treatment of the cultures with the different experimental factors tested in the current work was performed 24, 72, or 120 hours prior to cell harvesting based on the respective half-maximal inhibitory concentrations (IC_50_). Metabolic inhibitors were administered at various concentrations during differentiation: (A) ATR: 1 day—100 *μ*M, 3 days—46 *μ*M, and 5 days—36 *μ*M; (B) M*β*CD: 1 day—2.7 mM, 3 days—1.9 mM, and 5 days—1.1 mM; (C) SIM: 1 day—125 *μ*M, 3 days—10 *μ*M, and 5 days—7.5 *μ*M; (D) geranylgeranyltransferase inhibitor GGTI-286: 1 day—25 *μ*M, 3 days—24 *μ*M, and 5 days—23 *μ*M, respectively. The results are indicative of five independent experiments performed in eight replicates. Supplementary Data 2: muscle cell nuclei were stained with bisbenzimide (HO 33342) and imagined with ultraviolet light using the common DAPI filter with mercury-arc lamp of the fluorescent microscope. Presence of apoptotic nuclei was evaluated by nuclear shrinkage and chromatin condensation (see Materials and Methods). Horizontal panels of photographs from top to bottom: CTRL: control, CTRL + DMSO: vehicle control, ATR: atorvastatin (1 day—100 *μ*M, 3 days—46 *μ*M, and 5 days—36 *μ*M), SIM: simvastatin (1 day—125 *μ*M, 3 days—10 *μ*M, and 5 days—7.5 *μ*M), M*β*CD: methyl-*β*-cyclodextrin (1 day—2.7 mM, 3 days—1.9 mM, and 5 days 1.1 mM), MEV: mevalonate (100 *μ*M), GGOH: geranylgeraniol (10 *μ*M), FOH: farnesol (10 *μ*M), Chol-PEG: water-soluble cholesterol (1 mM) in combined treatments. Magnification 1 × 20000. Supplementary Data 3: acidic vacuolar organelle vital staining with acridine orange (AO). AVO were imagined with blue light using the green filter in the fluorescent microscope. AVO are present as yellow to red granules to demonstrate autophagy in muscle cells (see Materials and Methods). Horizontal panels of photographs from top to bottom: CTRL: control, CTRL + DMSO: vehicle control, ATR: atorvastatin (1 day—100 *μ*M, 3 days—46 *μ*M, and 5 days—36 *μ*M), SIM: simvastatin (1 day—125 *μ*M, 3 days—10 *μ*M, and 5 days—7.5 *μ*M), M*β*CD: methyl-*β*-cyclodextrin (1 day—2.7 mM, 3 days—1.9 mM, and 5 days—1.1 mM), MEV: mevalonate (100 *μ*M), GGOH: geranylgeraniol (10 *μ*M), FOH: farnesol (10 *μ*M), Chol-PEG: water-soluble cholesterol (1 mM) in combined treatments. Magnification 1 × 20000. Supplementary Data 4: myotube formation from C2C12 myoblasts. Myosin sarcomere expression was envisaged by cytoimmunofluorescence. Myosin is visible in green and nuclei in blue (see Materials and Methods). Monolayers were photographed at days 1, 3, and 5, of differentiation process. Horizontal panels of photographs from top to bottom: muscle cell phenotype was monitored in phase-contrast microscope (magnification 1 × 5000). Monolayers were photographed at days 1, 3, and 5, of differentiation process. Horizontal panels of photographs from top to bottom: CTRL: control, CTRL + DMSO: vehicle control, ATR: atorvastatin (1 day—100 *μ*M, 3 days—46 *μ*M, and 5 days—36 *μ*M), SIM: simvastatin (1 day—125 *μ*M, 3 days—10 *μ*M, and 5 days—7.5 *μ*M), M*β*CD: methyl-*β*-cyclodextrin (1 day—2.7 mM, 3 days—1.9 mM, and 5 days—1.1 mM), MEV: mevalonate (100 *μ*M), GGOH: geranylgeraniol (10 *μ*M), FOH: farnesol (10 *μ*M), Chol-PEG: water-soluble cholesterol (1 mM) in combined treatments. Magnification 1 × 10000.
