# Supplementary figures and images for: Geranylgeraniol Prevents Statin-Dependent Myotoxicity in C2C12 Muscle Cells through RAP1 GTPase Prenylation and Cytoprotective Autophagy
Source: Oxid Med Cell Longev. 2018 May 21;2018:6463807. doi: 10.1155/2018/6463807 (PMC5987243; doi:10.1155/2018/6463807)

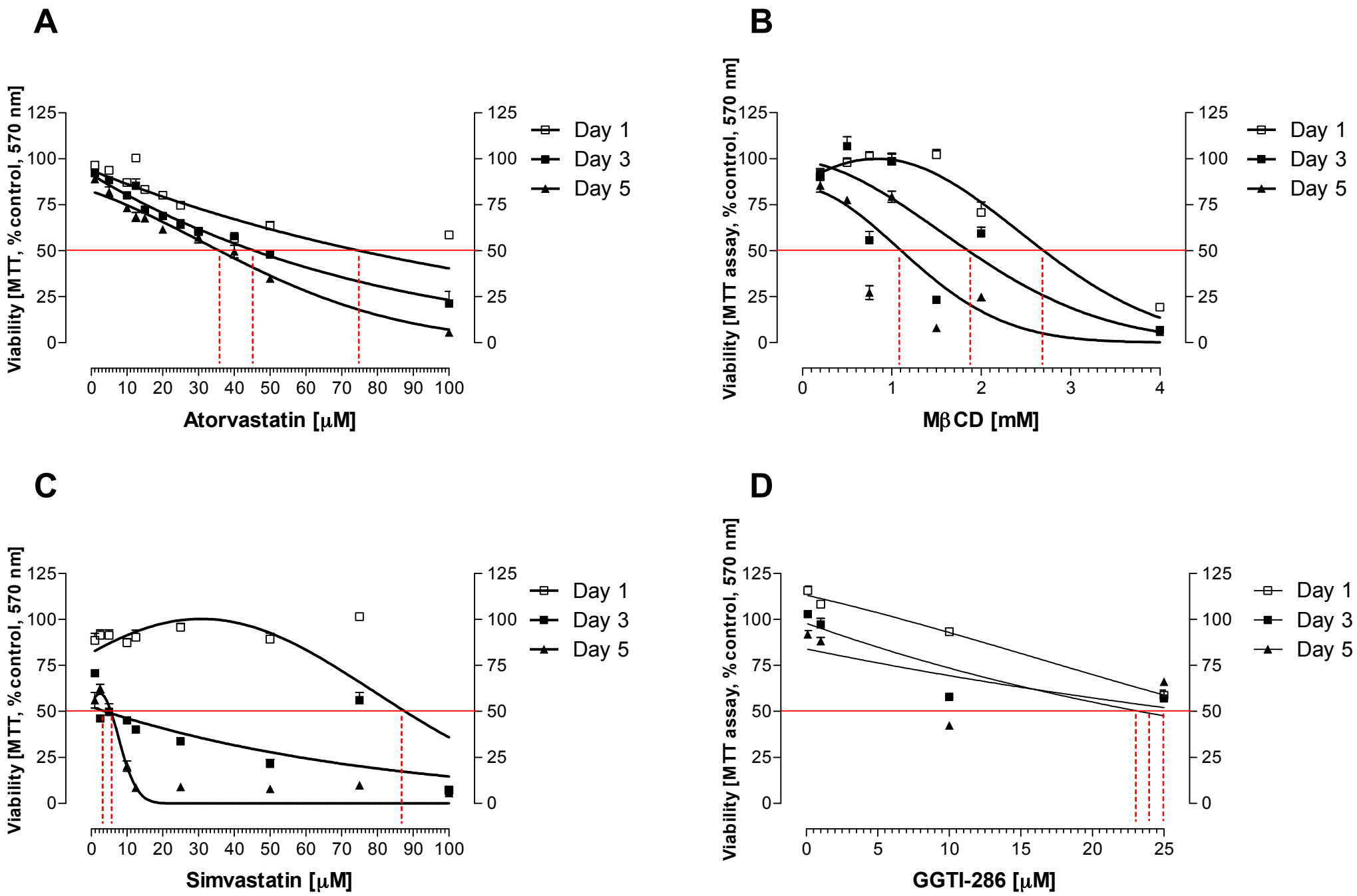

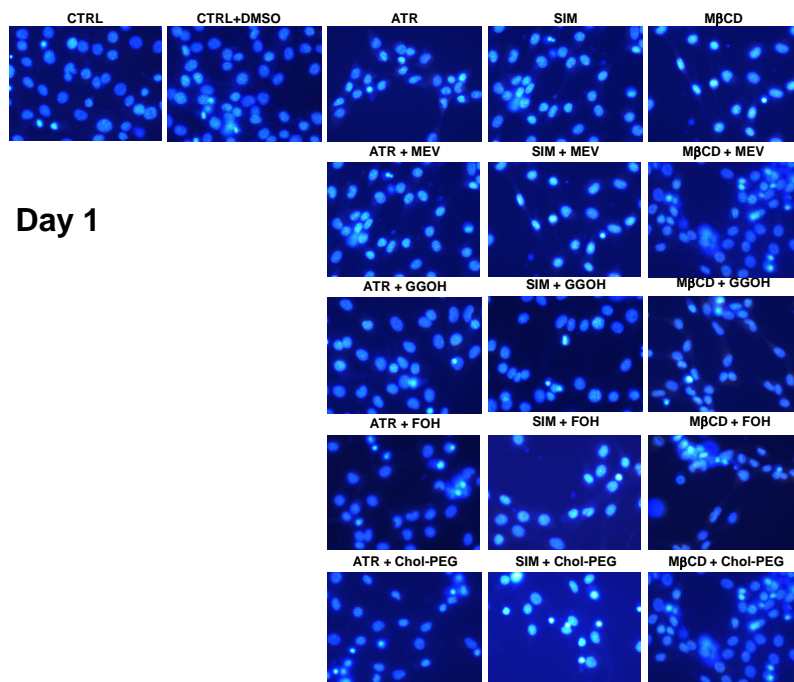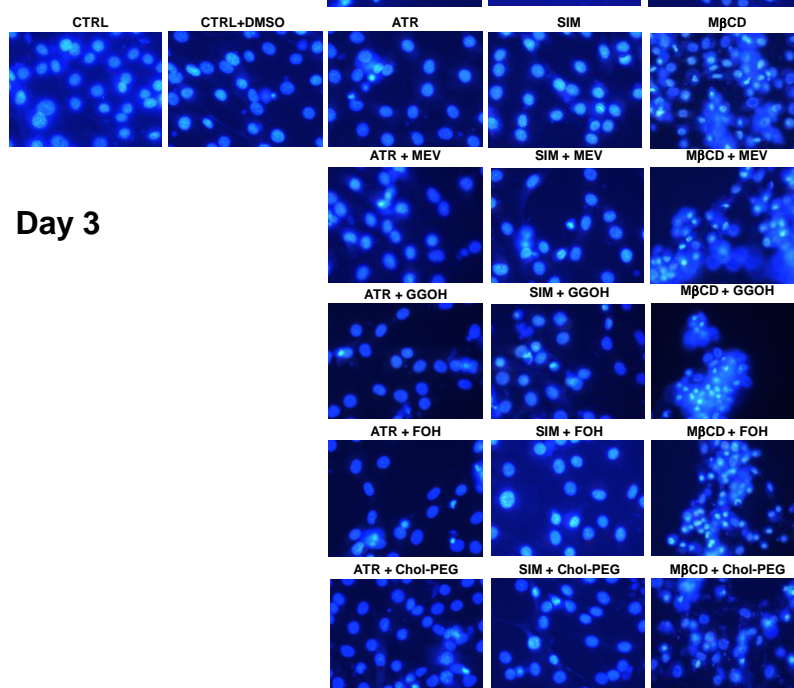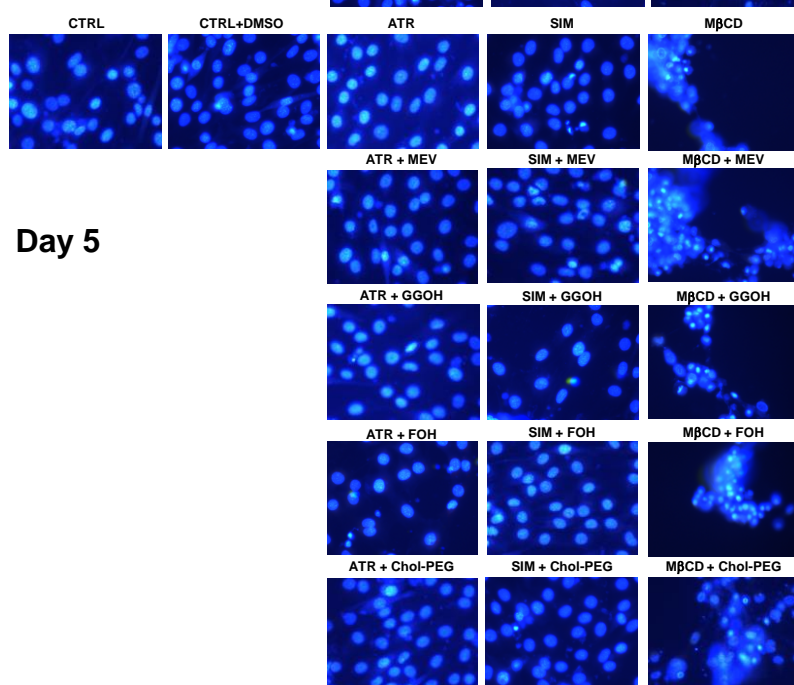

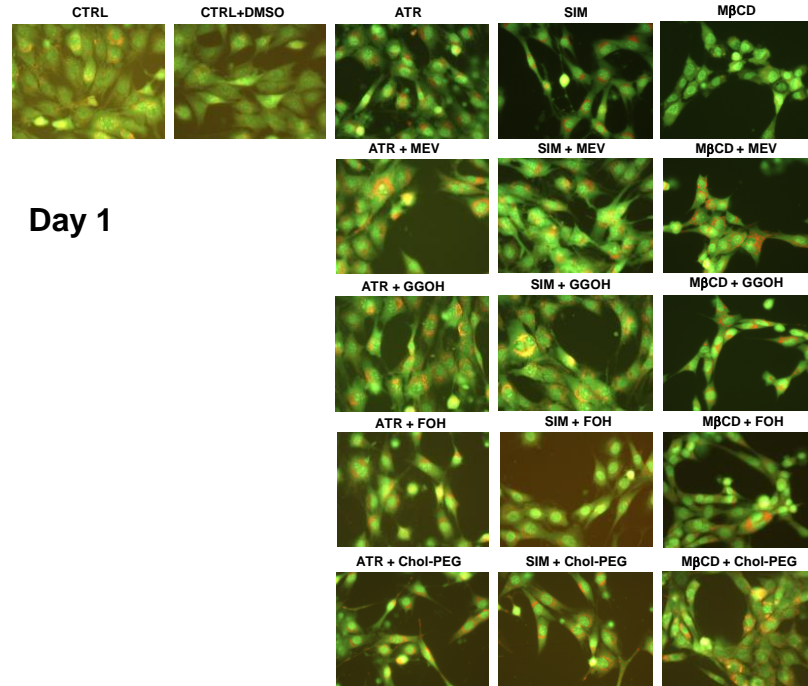

Day 1

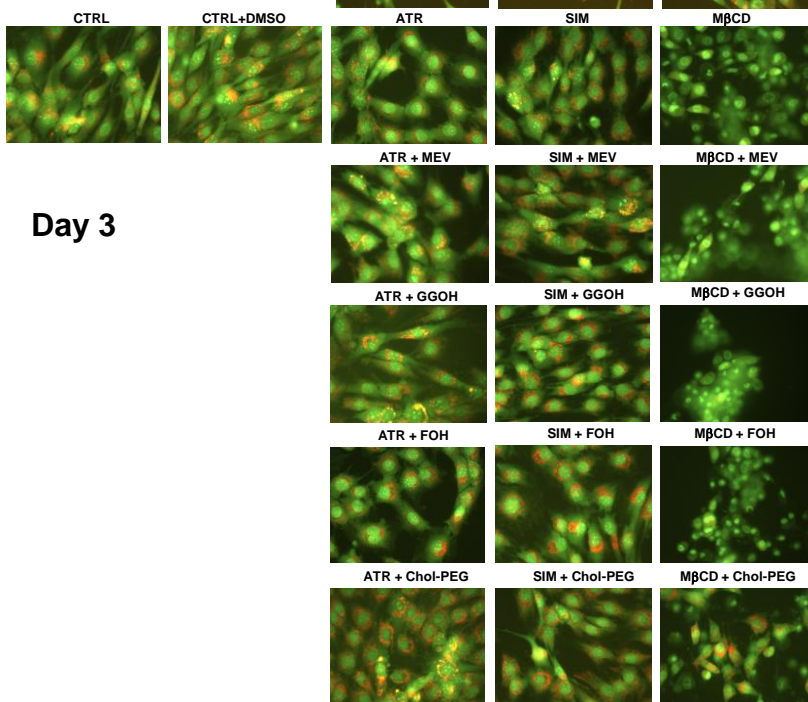

Day 3

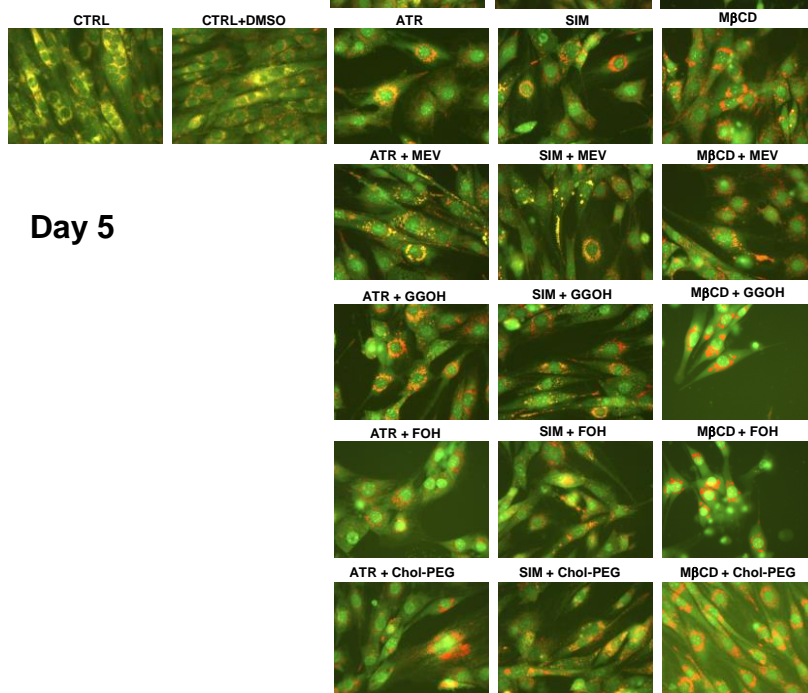

Day 5

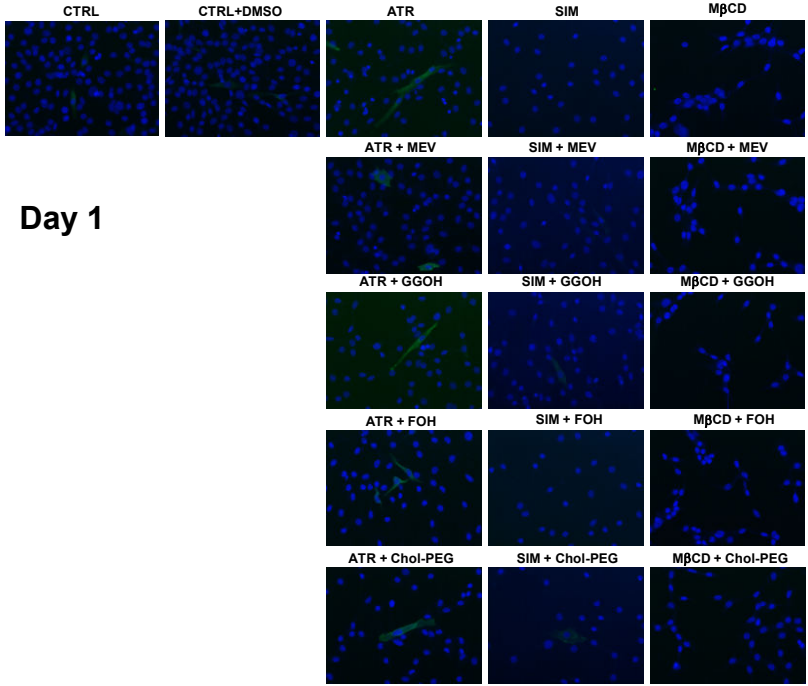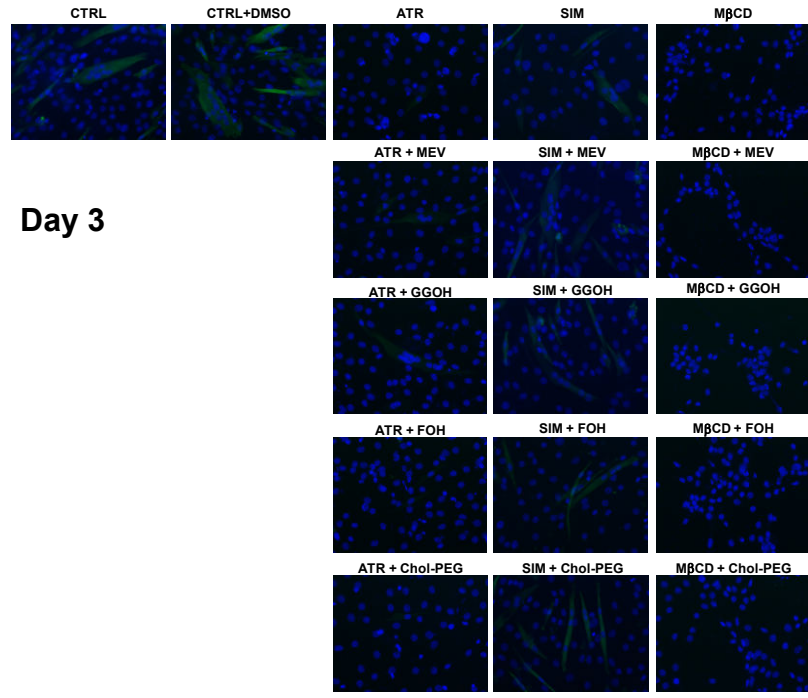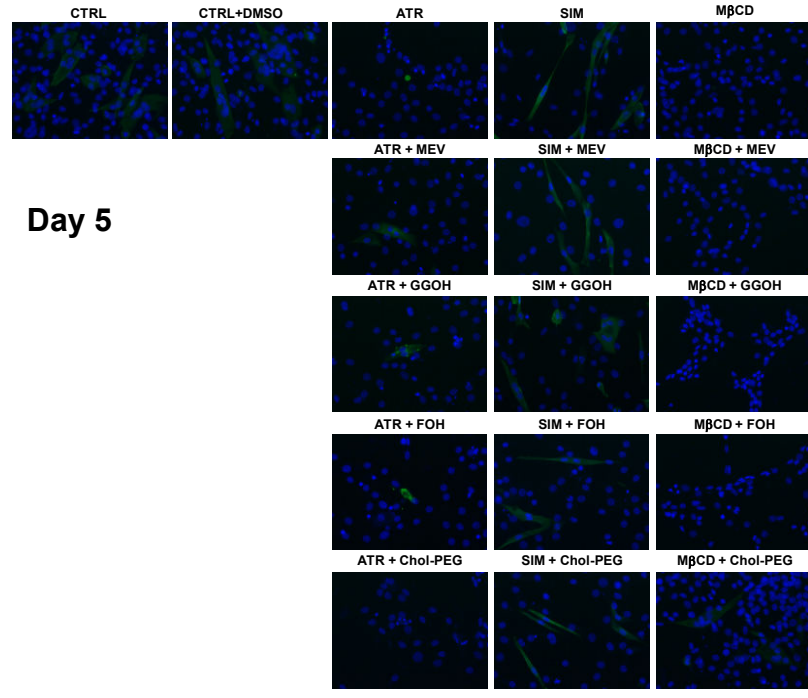

Supplement: Supplementary Materials — Supplementary Data 1: establishing the half-maximal inhibitory concentrations (IC50). Preliminary experiments were carried out with increasing concentrations of metabolic inhibitors in order to establish IC50 concentration (the half-maximal inhibitory concentration IC50 is a measure of the effectiveness of a substance in inhibiting a specific biological or biochemical function). A series of concentrations for statins (0–100 μM) were used to determine cell viability in the subsequent days of myogenesis (day 1—proliferating myoblasts, day 3—differentiating myotubes, and day 5—differentiated myotubes). Dose-response curves represent least squares of nonlinear Gaussian distribution. The IC50 values of factor for each day (1, 3, and 5) were found by extrapolation 50% cell viability (red dotted line) to the horizontal coordinate (abscissa). Treatment of the cultures with the different experimental factors tested in the current work was performed 24, 72, or 120 hours prior to cell harvesting based on the respective half-maximal inhibitory concentrations (IC50). Metabolic inhibitors were administered at various concentrations during differentiation: (A) ATR: 1 day—100 μM, 3 days—46 μM, and 5 days—36 μM; (B) MβCD: 1 day—2.7 mM, 3 days—1.9 mM, and 5 days—1.1 mM; (C) SIM: 1 day—125 μM, 3 days—10 μM, and 5 days—7.5 μM; (D) geranylgeranyltransferase inhibitor GGTI-286: 1 day—25 μM, 3 days—24 μM, and 5 days—23 μM, respectively. The results are indicative of five independent experiments performed in eight replicates. Supplementary Data 2: muscle cell nuclei were stained with bisbenzimide (HO 33342) and imagined with ultraviolet light using the common DAPI filter with mercury-arc lamp of the fluorescent microscope. Presence of apoptotic nuclei was evaluated by nuclear shrinkage and chromatin condensation (see Materials and Methods). Horizontal panels of photographs from top to bottom: CTRL: control, CTRL + DMSO: vehicle control, ATR: atorvastatin (1 day—100 μM, 3 days—46 μM, and 5 [file 6463807.f1.pdf]
